# Activation of the *Arabidopsis thaliana* Immune System by Combinations of Common *ACD6* Alleles

**DOI:** 10.1371/journal.pgen.1004459

**Published:** 2014-07-10

**Authors:** Marco Todesco, Sang-Tae Kim, Eunyoung Chae, Kirsten Bomblies, Maricris Zaidem, Lisa M. Smith, Detlef Weigel, Roosa A. E. Laitinen

**Affiliations:** Department of Molecular Biology, Max Planck Institute for Developmental Biology, Tübingen, Germany; Duke University, United States of America

## Abstract

A fundamental question in biology is how multicellular organisms distinguish self and non-self. The ability to make this distinction allows animals and plants to detect and respond to pathogens without triggering immune reactions directed against their own cells. In plants, inappropriate self-recognition results in the autonomous activation of the immune system, causing affected individuals to grow less well. These plants also suffer from spontaneous cell death, but are at the same time more resistant to pathogens. Known causes for such autonomous activation of the immune system are hyperactive alleles of immune regulators, or epistatic interactions between immune regulators and unlinked genes. We have discovered a third class, in which the *Arabidopsis thaliana* immune system is activated by interactions between natural alleles at a single locus, *ACCELERATED CELL DEATH 6* (*ACD6*). There are two main types of these interacting alleles, one of which has evolved recently by partial resurrection of a pseudogene, and each type includes multiple functional variants. Most previously studies hybrid necrosis cases involve rare alleles found in geographically unrelated populations. These two types of *ACD6* alleles instead occur at low frequency throughout the range of the species, and have risen to high frequency in the Northeast of Spain, suggesting a role in local adaptation. In addition, such hybrids occur in these populations in the wild. The extensive functional variation among *ACD6* alleles points to a central role of this locus in fine-tuning pathogen defenses in natural populations.

## Author Summary

Plants and their pathogens are engaged in an endless evolutionary battle. The invention of new strategies by pathogens pushes plants to continuously update their defenses. This in turn leads the pathogens to circumvent these new defenses, and so on. Given the abundance of potential enemies, it is therefore not surprising that genes involved in defense against pathogens are among the most variable in plants. A drawback of this extreme variation in pathogen-recognition mechanisms is that at times the plant mistakes itself for an enemy, leading to autonomous activation of defense responses in the absence of pathogens. Conventional models for this phenomenon, called hybrid necrosis, require the interaction between two different genes. Here we show instead that hybrid necrosis can be triggered by interactions between variants of a single gene, *ACD6* (*ACCELERATED CELL DEATH 6*). Several of these variants are common in natural *Arabidopsis thaliana* populations and can interact to give different levels of activation of the immune system. Our results provide important information into the evolution and operation of the plant defense system. Moreover, the abundant presence of *ACD6* functional variation suggests a major role for this gene in modulating plant defenses in nature.

## Introduction

Despite its inherent advantage, resistance to pathogens is highly variable in natural populations [1]. One explanation for this lies in fluctuating pathogen pressures, which are expected to result in fitness tradeoffs between maintaining continuous defenses and the metabolic costs incurred in the absence of enemies [2]–[4]. An alternative explanation for individual differences in disease resistance comes from the evershifting front in the evolutionary arms race between pathogens and their hosts. Accordingly, immunity loci are among the most variable genes in both animal and plant genomes [5]–[7]. Yet, too much variation can be dangerous, and lead to inadvertent self-recognition and autoimmunity.

Autoactivation of defenses in the absence of pathogens has been observed both in inbred strains and in hybrid progeny. One of the most visible outcomes of this phenomenon is widespread necrosis due to extensive cell death in leaves, mimicking the hypersensitive response (HR) that is often mounted upon pathogen attack [8]. The most severe cases are those reported in intra- and interspecific plant hybrids. Most cases of hybrid necrosis have been identified in controlled crosses, but some occur in nature [9], [10].

About 2% of random crosses between wild strains (accessions) of *Arabidopsis thaliana* (henceforth Arabidopsis) result in F_1_ plants that are smaller than their parents and that have overt signs of leaf necrosis [11]. As in other species [8], [12]–[14], the causal genes identified so far encode either immune receptors or regulators of the immune response [11], [15]. Similar, but weaker symptoms are seen in inbred strains that carry a naturally occurring hyper-active allele of the *ACCELERATED CELL DEATH 6* (*ACD6*) gene; like necrotic hybrids [11], these plants show enhanced resistance to pathogens, but suffer from compromised growth [16].

Here we report a new phenomenon, single-locus hybrid necrosis. Several special alleles at the *ACD6* locus interact to activate the immune system independently of the presence of pathogens. The increased immunity in these hybrids is associated with a temperature-dependent reduction in size and fertility. These alleles are responsible for several cases of hybrid necrosis observed in controlled crosses, and, unlike the causal alleles for other cases of hybrid necrosis in Arabidopsis [11], [15], they are common and co-occur in nature. The causal alleles themselves are heterogeneous, and interactions between different combinations elicit different levels of defense responses. Furthermore, the high frequencies of these alleles in the Costa Brava region of Northeastern Spain suggest that they play a role in local adaptation.

## Results

### Interactions between *ACD6* alleles causing hybrid necrosis

**Figure 1:**
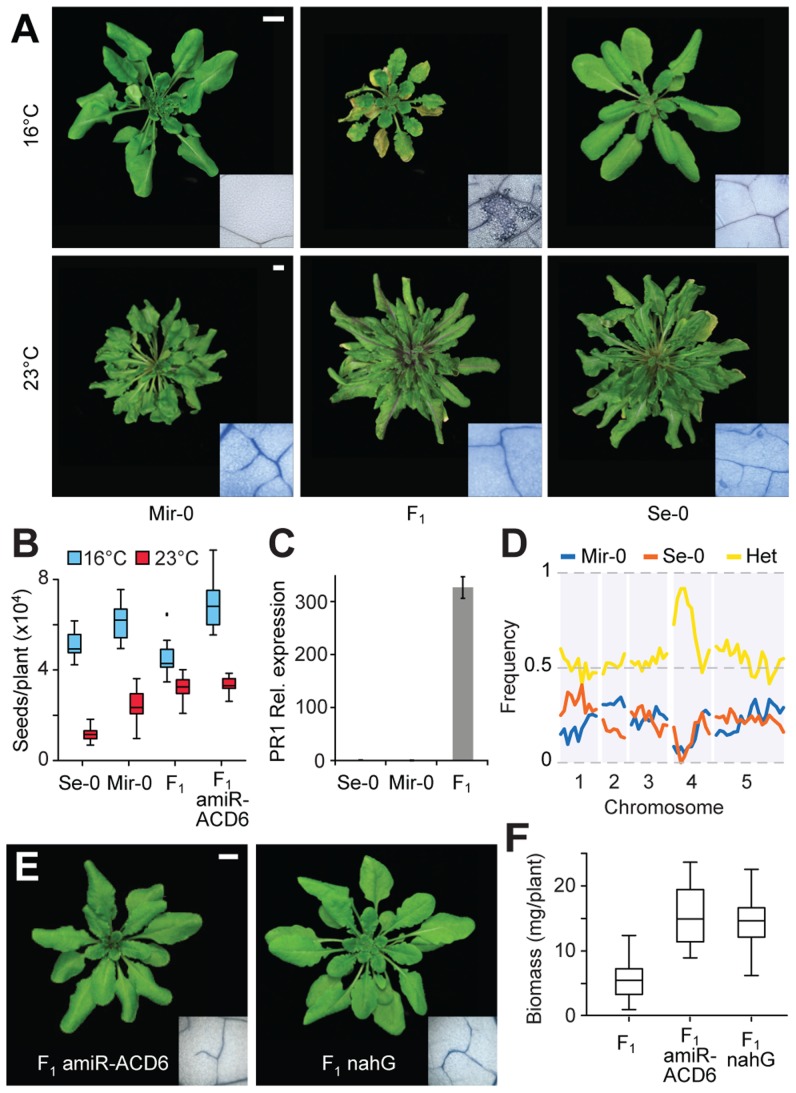
Identification of *ACD6* as causal for Mir-0×Se-0 temperature-sensitive hybrid necrosis. (**A**) Rosettes of a six-week-old Mir-0 and Se-0 plants and their F_1_ hybrid. Insets show leaves stained with Trypan blue for dead cells. (**B**) Seed set. Averages for 15 individuals are shown. Seed set is significantly different between F_1_ and F_1_ amiR-ACD6 plants at 16°C (p<0.001), but not at 23°C (p = 0.69). (**C**) Relative expression levels of *PR1*, as measured by quantitative RT-PCR (averages for five biological replicates). Expression values were normalized to those of Se-0 individuals. Differences in *PR1* expression between Mir-0×Se-0 hybrids and the parental genotypes were significant at p<0.05. Error bars represent standard errors. (**D**) Genome-wide mapping of the hybrid necrosis phenotype in an F_2_ population. (**E**) Rosettes of six-week-old hybrids grown at 16°C, with necrosis suppressed by *ACD6* knockdown or nahG-mediated depletion of SA (see the non-transgenic hybrid grown at 16°C in Figure 1A for comparison). Insets show leaves stained with Trypan blue for dead cells. (**F**) Aboveground biomass for six-week-old plants grown at 16°C. Averages for at least 10 individuals are shown. Size bars = 1 cm.

Hybrid necrosis cases in Arabidopsis differ in their severity, with some hybrids dying, while others are merely dwarfed. One of the mildest examples is provided by a cross between the Mir-0 and Se-0 accessions from Miramare in Italy and San Elano in Spain [11]. At 16°C, necrosis appeared after three to four weeks in older leaves of Mir-0×Se-0 hybrids, and both final size and fertility were reduced compared to their parents. As with other cases of hybrid necrosis in Arabidopsis, these phenotypes largely disappeared at 23°C [11] (Figure 1A,B). Expression of the disease resistance marker gene *PR1* was elevated in the hybrids (Figure 1C), consistent with their increased resistance to the pathogen *Hyaloperonospora arabidopsidis* [11]. Importantly, resistance has been observed at temperatures that suppress the morphological defects [11].

Genetic mapping in the F_2_ generation established that the necrotic phenotype of Mir-0×Se-0 hybrids was linked to a single region of the genome (Figure 1D), in agreement with a 1∶1 segregation ratio of normal and F_1_-like plants in the F_2_ generation [11]. The final mapping interval of 290 kb (between 8.11 and 8.40 Mb on chromosome 4) included three immunity loci: At4g14370, which encodes an immune receptor of the TIR-NBS-LRR type; *ACD6* (At4g14400); and the adjacent *ACD6* paralog At4g14390. We knocked down each gene with artificial microRNAs (amiRNAs; [17]). Only the amiRNA against *ACD6* suppressed leaf necrosis and increased plant size and seed production in hybrids (Figure 1B,E,F; Figure S1). *ACD6* encodes an ankyrin repeat transmembrane protein that acts mainly through the hormone salicylate (SA) [16], [18]–[20]. In agreement, depletion of SA by expression of a bacterial salicylate hydroxylase, nahG [21], resulted in suppression of the hybrid phenotypes as well (Figure 1E,F).

**Figure 2:**
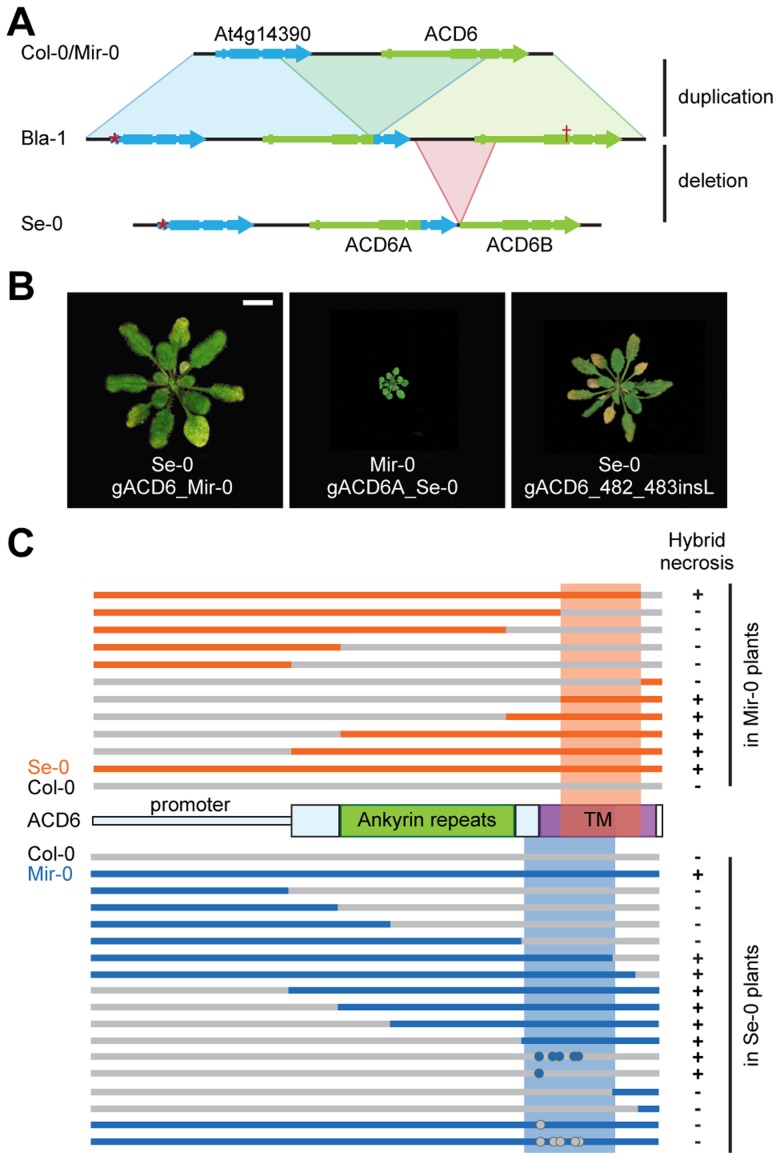
Mapping of causal regions in *ACD6* alleles. (**A**) Organization of the *ACD6* locus in Col-0, Bla-1 and Se-0. Col-0 likely represents the ancestral configuration of the *ACD6* region. A partial duplication of At4g14390 and *ACD6* followed by a deletion of the 5′ portion of *ACD6B* likely produced first the Bla-1 and then the Se-0 arrangement. Asterisk indicates missing start codon in At4g14390, the cross a premature stop codon in the Bla-1 allele of *ACD6B*. Size bar = 1 cm. (**B**) Rosettes of six-week-old plants in which transgenic introduction of *ACD6* alleles recapitulated the phenotype of hybrids. The gACD6_482_483insL transgene is the Col-0 allele of *ACD6* with a single amino acid insertion between position 482 and 483. (**C**) Schematic representation of chimeric constructs used to map *ACD6* sequences causal for hybrid necrosis. Dots indicate single amino acid changes.

The *ACD6* locus of Mir-0 had a similar organization as the reference Col-0 allele (Figure 2A), and a 7.2 kb genomic fragment spanning *ACD6* reproduced the hybrid phenotype when transformed into Se-0 plants (Figure 2B, Table S1). In Se-0, there were two tandem copies of *ACD6* (*ACD6A* and *ACD6B*; Figure 2A); only transformation of *ACD6A* into Mir-0 caused necrosis and reduced growth (Figure 2B, Table S1). Similar transgenic experiments with Col-0 and its *ACD6* allele confirmed the specificity of the interaction between the Mir-0 and Se-0 alleles of *ACD6* (Table S1).

Experiments with chimeric transgenes showed that promoter activity did not account for differences between Mir-0, Se-0 and Col-0 alleles (Figure 2C). Domain swaps and site-directed mutagenesis further localized residues responsible for hybrid necrosis to the transmembrane domain for both the Mir-0 and Se-0 alleles (Figure 2C). These experiments point to the interallelic interaction occurring at the protein level.

**Figure 3:**
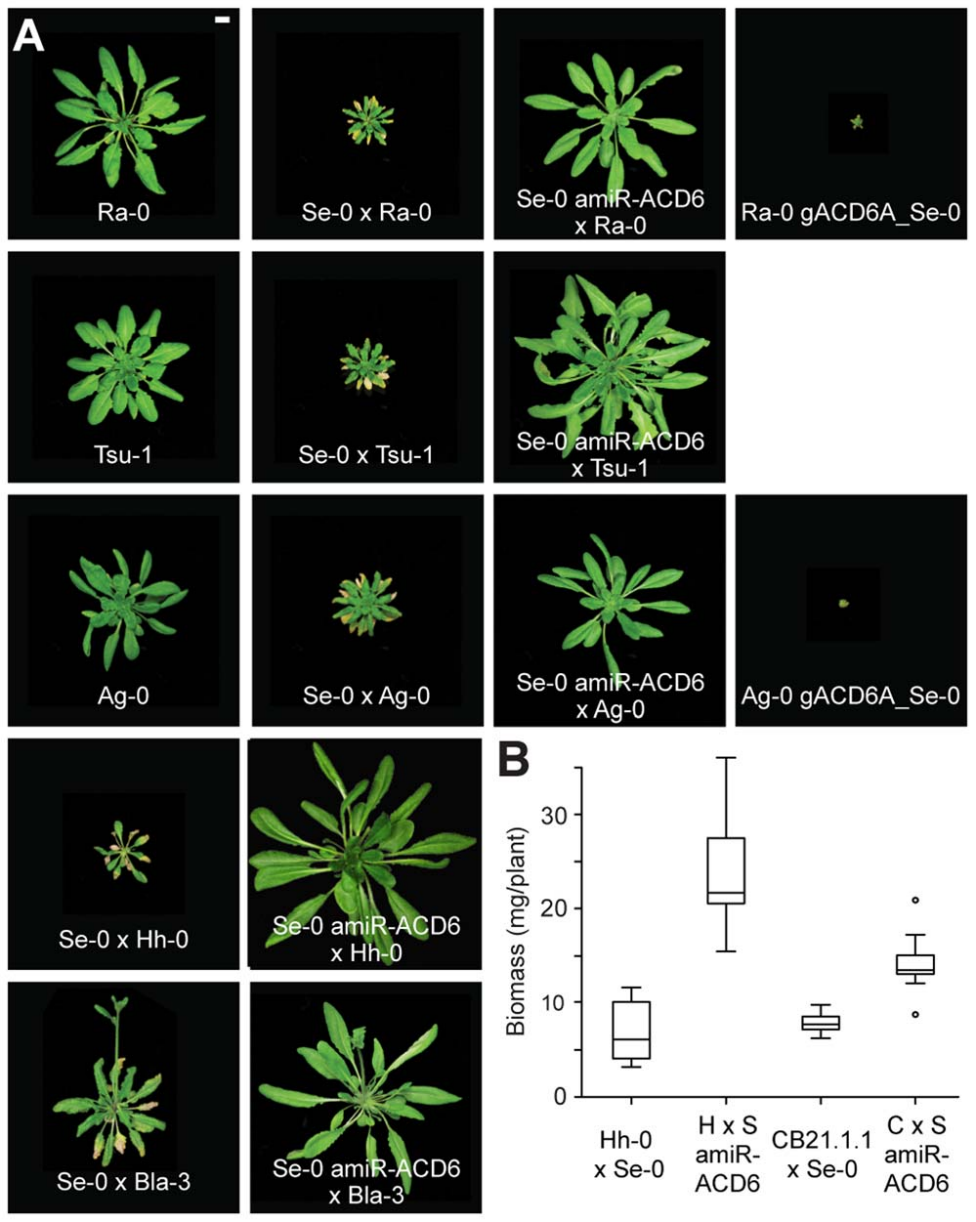
Lesions and reduced biomass in different hybrids. (**A**) Rosettes of six-week-old plants. Size bar = 1 cm. (**B**) Aerial biomass for six-week-old plants grown at 16°C. Averages for at least eight individuals are shown. CB21.1-1 carries a Mir-0-like class III allele of *ACD6*, and crosses with Se-0 resulted in milder necrosis than for accessions carrying a Mir-0-like class I or II allele. Accordingly, the effect of knocking down *ACD6* in these hybrids had a smaller effect on biomass production. Differences between transgenic lines and the respective hybrids were significant at p<0.001.

Since the first report of hybrid necrosis in Arabidopsis [11], we have identified additional examples of hybrid necrosis, including several other Mir-0×Se-0-like cases (Table 1; Tables S2, S3). Using test crosses, segregation analyses, amiRNA knockdowns, and transformation with genomic fragments from Mir-0 and Se-0, we confirmed Mir-0- and Se-0-like alleles of *ACD6* as causal for several independent hybrid cases (Table S1, S2; Figure 3). We compared *ACD6* sequences between these and other Arabidopsis strains to gain a better understanding of the activity and evolutionary background of these alleles. Notably, while Mir-0-like strains had a broad distribution that included much of the native range of the species throughout Eurasia, Se-0-like strains were only found in the Northeast of Spain, along the Costa Brava (Table 1).

**Table 1:** Accessions with *ACD6* hybrid necrosis alleles (see also Tables S1 and S2).

Class	Accession	Origin	Original Evidence
**Mir-0-like, class I**	Hh-0	Germany	cross
Severe with Se-0; mild with Bla-1	ICE79	Italy	cross
	Ag-0	France	sequence
	SP5.7-1	Spain (Costa Brava)	sequence
	TOU-A1-96	France	haplotype
	LAC3	France	haplotype
	TDr-1	Sweden	haplotype
**Mir-0-like, class II**	Mir-0	Italy	cross
Severe with Se-0; none with Bla-1	Bla-3	Spain (Costa Brava)	cross
	Ws	Russia	cross
	Er-0	Germany	cross
	Ra-0	France	sequence
	Tsu-1	Italy	sequence
	Ler-1	Germany	sequence
	Omo2-1	Sweden	sequence
	Ws-0	Russia	sequence
	Belmonte4-94	Italy	haplotype
**Mir-0-like, class III**	C24	Portugal	cross
Mild with Se-0; none with Bla-1	CB16-2	Spain (Costa Brava)	sequence
	CB17-3	Spain (Costa Brava)	sequence
	CB17-5	Spain (Costa Brava)	sequence
	CB21.1-1	Spain (Costa Brava)	sequence
**Se-0-like**	Se-0	Spain (Costa Brava)	cross
Severe with Mir-0-like classes I and II; mild with Mir-0-like class III	Pla-1	Spain (Costa Brava)	cross
	Sf-2	Spain (Costa Brava)	cross
	CB5-4	Spain (Costa Brava)	sequence
	CB6-1	Spain (Costa Brava)	sequence
	CB15-1	Spain (Costa Brava)	sequence
	CB17-12	Spain (Costa Brava)	sequence
	CB22-3	Spain (Costa Brava)	sequence
**Bla-1-like**	Bla-1	Spain (Costa Brava)	cross
Mild with Mir-0-like class I	CB5-4	Spain (Costa Brava)	sequence
	UKSE06-520	UK	haplotype
	ROM-1	France	haplotype

### A complex evolutionary history for Se-0-like alleles

**Figure 4:**
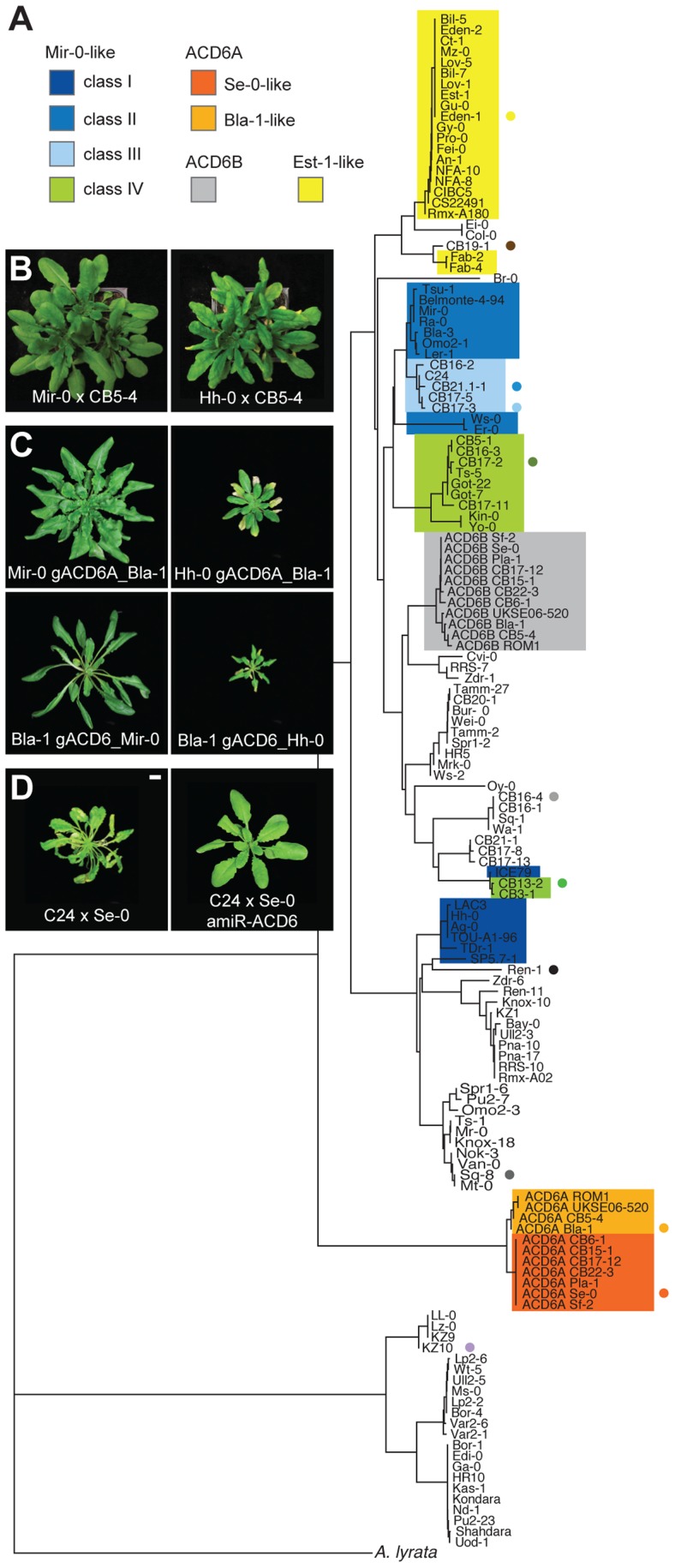
Functional diversity of the *ACD6* alleles. (**A**) Hierarchical clustering of *ACD6* alleles from 131 Arabidopsis accessions and the *Arabidopsis lyrata* strain MN47. Classes are described in the text and in Table 1. Dots indicate genotypes found at the CB16 collection site near Llagostera, Spain (see Fig. 5B). The deeply diverged group above the *A. lyrata* MN47 allele is the KZ-10 group (described in [16]). (**B**) Rosettes of six-week-old plants grown at 16°C. The CB5-4 accession carries a Bla-1-like allele of *ACD6*, and produced mild necrosis only when crossed to Hh-0-like accessions. Crosses with CB5-4 instead of Bla-1 are shown because an additional, genetically independent mechanism of incompatibility between Bla-1 and Hh-0 complicated observation of the necrosis phenotype in these crosses. (**C**) Rosettes of six-week-old plants transformed with different genomic constructs and grown at 16°C. (**D**) Rosettes of six-week-old plants grown at 16°C. C24 has a Mir-0-like class III *ACD6* allele; interaction with the Se-0 allele resulted in mild hybrid necrosis. Size bars = 1 cm.

As described above, the *ACD6* locus in Se-0 contained an additional *ACD6* copy. This organization is most likely derived, since we did not find it in other Arabidopsis strains or in *Arabidopsis lyrata*. The *ACD6* paralog At4g14390, located immediately upstream of *ACD6*, lacked a start codon in Se-0, while a fragment from the promoter through part of the first intron was missing in *ACD6B*, indicating that only one of the three genes, *ACD6A*, was functional. *ACD6A* appeared to be a chimeric gene that formed recently through intralocus duplication and recombination, deduced from the 3′ portion being almost identical to that of the upstream At4g14390 gene, and the 5′ region from the first intron on being almost identical to that of the downstream *ACD6B* gene (Figure 2A). Thus, the *ACD6A* sequences causal for hybrid necrosis were derived from the At4g14390 pseudogene, Se-0-like alleles of At4g14390 were found in other strains with the ancestral two-gene organization of the *ACD6* locus (Figure 4A, Figure S2A), suggesting that the pseudogenized state of At4g14390 preceded the duplication event.

Across seven Se-0-like strains examined in detail, the entire 14 kb *ACD6* locus lacked any polymorphisms, supporting a very recent origin of this allele in Northeastern Spain or a recent selective sweep in this region. The Bla-1 strain, also from this region, had an arrangement that likely reflects the ancestral state with respect to the Se-0 allele, lacking the partial deletion of *ACD6B* (Figure 2A). Bla-1 crosses, however, produced hybrid necrosis only in combination with two of the Mir-0-like strains, Hh-0 and ICE79 (Figure 4B; Table 1; Table S2). Although *ACD6B* was expressed in Bla-1 (Figure S2B), a premature stop codon was predicted to truncate the open reading frame. The encoded protein lacks therefore most of the ankyrin repeats along with the transmembrane domain required for ACD6 activity [22] (Figure 2A). Few additional derived polymorphisms distinguished the Se-0-like from the Bla-1-like alleles. Transgenic experiments confirmed that *ACD6A* was causal for Bla-1×Hh-0 hybrid necrosis. They also showed that the five non-synonymous SNPs distinguishing the Se-0 and Bla-1 alleles of *ACD6A* were responsible for the failure of the Bla-1 allele to interact with the Mir-0 allele (Figure 4C; Table S1).

### Extensive sequence and functional variation in Mir-0-like alleles

In contrast to the Se-0-like alleles, there was considerable sequence variation among Mir-0-like alleles from Bla-3, Er-0, Hh-0, ICE79, and Ws-0 (Figure 4A), suggesting an older origin. *ACD6* alleles from these strains shared four characteristic amino acid substitutions and a single amino acid insertion. An *ACD6* transgene with the single amino acid insertion, a leucine between positions 482 and 483 (482_483insL), introduced into the Col-0 reference allele was sufficient to induce hybrid necrosis-like symptoms in Se-0 plants (Figure 2B, C; Figure S3), but only the Hh-0, and not the Mir-0 *ACD6* transgene recapitulated the hybrid phenotype in the Bla-1 background (Figure 4C), indicating that variation in the sequence of *ACD6* is responsible for the differences in the behavior of Mir-0-like accessions.

In agreement with Mir-0-like alleles being more broadly distributed, we found several additional strains with the Mir-0 causal polymorphism among a commonly used reference set of 96 strains [23] (Table 1; Figure 4A). Based on the phenotype of hybrids with Se-0 and Bla-1, we could divide these accessions into four classes. The first two were defined by the alleles described above: class I alleles, such as Hh-0, produced severely affected hybrids regardless of the crossing partner, while class II alleles, including Mir-0, interacted only with Se-0. Class III alleles also interacted only with Se-0, but produced milder symptoms compared to class II alleles. Finally, class IV alleles did not result in any necrosis (Figure 4A, D; Table 1; Tables S1, S2; Figure S4). The class III and IV alleles were distinguished from each other and from class I and II alleles by unique polymorphisms, suggesting that the reason for their different behavior resided in the *ACD6* gene itself. F_2_ progeny involving class III and IV alleles did not produce additional phenotypic classes either, confirming the absence of independently segregating, extragenic suppressors of necrosis (Table S3), and supporting functional differentiation among *ACD6* alleles.

### Local co-occurrence of hybrid necrosis *ACD6* alleles in Northeast Spain

Based on shared single nucleotide polymorphisms (SNPs) across the *ACD6* region, we identified additional Mir-0-like strains in a set of 1,307 unique accessions that had been genotyped at high density [24]. This set included eight known Mir-0-like strains and four known Se-0/Bla-1-like strains. Sixty-eight additional accessions had patterns of polymorphism consistent with Mir-0-like class I, II or III alleles. Sequence analysis of 25 that were representative for different subgroups indicated that all carried a hybrid necrosis-inducing *ACD6* allele. Test crosses with four of the new accessions confirmed that these alleles could induce hybrid necrosis in combination with Se-0 or Bla-1. At the same time, we found only four additional accessions with Se-0/Bla-1-like *ACD6* alleles in this global sample. All four came from outside Spain and had Bla-1-like sequence and activity, interacting only with Hh-0 and not Mir-0 (Table 1, Tables S1, S2 and S4).

**Figure 5:**
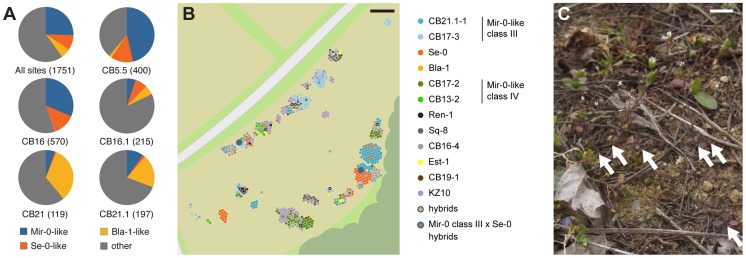
Co-occurrence of different *ACD6* alleles in the Costa Brava region. (**A**) *ACD6* allele frequencies in different Costa Brava populations. The numbers of genotyped individuals are given in parentheses. “Other” alleles include Mir-0-like class IV alleles, which do not induce hybrid necrosis. (**B**) Distribution of individuals carrying different *ACD6* alleles at the CB16 collection site near Llagostera, Spain. Alleles are named according to their similarity to those reported in Fig. 4A. (**C**) Example of Arabidopsis plants growing at site CB16. Arrows point to rosettes. Size bars = 10 m in B, 1 cm in C.

Because Mir-0- and Se-0/Bla-1-like alleles co-occurred in the Northeast of Spain, we investigated the distribution of *ACD6* alleles in this region in more detail. We first screened a set of 54 accessions with unique multi-locus genotypes collected in 2007 from the Costa Brava region (Table S5). We found that five each carried Mir-0- and Se-0/Bla-1-like *ACD6* alleles (Table 1). Crosses between these strains and with Mir-0, Hh-0, Se-0 and Bla-1 produced the expected phenotypes, as did *ACD6* knockdowns (Figure S4; Table S2). To further determine whether *ACD6* alleles responsible for hybrid necrosis co-occurred in local populations, we collected over 2,000 individuals representing 13 populations in March 2012 (Table S6). Sequencing of the *ACD6* locus in 1,751 of these samples [25] confirmed that these populations harbored both Mir-0- and Se-0/Bla-1-like alleles (Figure 5A). We focused on one population, CB16, with 640 individuals, in an uncultivated field on the immediate outskirts of Llagostera. Sequencing identified 12 different *ACD6* alleles in this population, including two distinct Mir-0-like class III alleles (combined frequency 31%) and an Se-0-like allele (frequency 13%) (Figure 5B). Despite Arabidopsis being predominantly selfing, moderate levels of outcrossing are frequently observed in natural populations [26], and 12 plants were heterozygous at the *ACD6* locus. Two of these hybrids were heterozygous for Mir-0- and Se-0-like alleles (Figure 5B).

**Figure 6:**
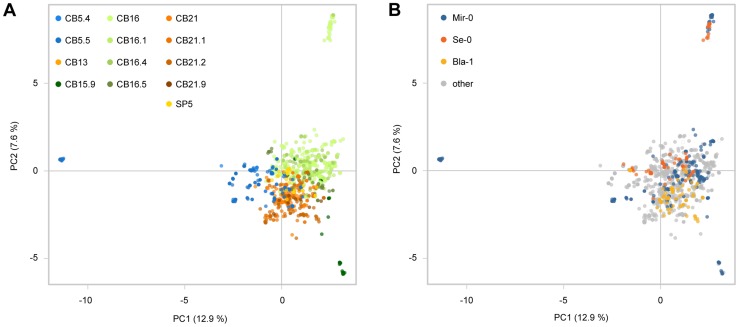
Genome-wide analysis of Costa Brava populations. (**A**) Principal component analysis (PCA) of 1,688 Costa Brava individuals with 730 SNP markers with complete information. Populations are color coded. (**B**) Same as in (A), but individuals are color coded by *ACD6* allele types.

We then analyzed 3,641 restriction site associated DNA (RAD) markers in 1,688 individuals from the Costa Brava region [25]. These included 147 individuals that shared the Se-0 allele at *ACD6*: despite the lack of diversity at *ACD6* in these individuals, there was substantial genome-wide diversity in this group (Figure 6), indicating that the Se-0 allele had not merely spread as a clonal lineage, but also through outcrossing. While linkage disequilibrium (LD) around *ACD6* tended to be generally lower than the genome-wide average, this was not the case for the Se-0 group, in agreement with a recent origin of the Se-0 allele (Figure S5). Mir-0-like alleles were found in Northeast Spain at a high frequency as well; out of 958 individuals with a unique multi-locus genotype, 22% (207) carried Mir-0-like alleles, while 9% (81) had an Se-0 allele and 7%(70) a Bla-1 allele. These percentages are significantly higher than in the global sample of Arabidopsis accessions (Mir-0-like = 6%; Se-0 = ND; Bla-1 = 0.3%; p<0.01).

## Discussion

### Hybrid necrosis caused by allelic interactions at a single locus

Hybrid necrosis has attracted the attention of plant breeders and evolutionary biologists for decades [8], not least because they conform to the classical two-gene models of Bateson, Dobzhansky and Muller for the evolution of genetic incompatibilities [11]–[15]. We find that interactions between two alleles of a single gene, *ACD6*, are sufficient to induce similar hyper-activation of the plant immune system, making this the first described case of single-locus hybrid necrosis.

### Insights into *ACD6* function from hybrid necrosis alleles

*ACD6* encodes a transmembrane protein with ankyrin repeats. In plants with the induced gain-of-function allele *acd6*-1 a single amino acid change in the transmembrane domain leads to constitutive activation of defense responses, resulting in increased pathogen resistance, extensive leaf necrosis and stunted growth [18], [19], [22]. We recently described a similarly hyper-active allele of *ACD6* in natural strains of Arabidopsis [16]. This allele, dubbed *ACD6*-Est from the name of the strain in which it was initially discovered, segregates at intermediate frequencies in the global Arabidopsis population, and strains carrying it display similar phenotypes as the *acd6*-1 mutant. Although *ACD6*-Est carries many different non-synonymous substitutions compared to the reference allele, only two amino acid changes in the transmembrane domain are required for its gain-of-function activity (Figure S3, Table S7).

Mir-0×Se-0 hybrids present more extreme phenotypes than strains with the *ACD6*-Est allele. Interactions between *ACD6* alleles in Mir-0×Se-0 hybrids occur at the protein level, which is consistent with ACD6 forming oligomeric complexes [27]. As with *acd6*-1 and *ACD6*-Est, the causal amino acid changes in the Mir-0 and Se-0 alleles map to the transmembrane domain (Figure S3, Table S7). These observations confirm the functional importance of the transmembrane domain and suggest a major role of multimerization in regulating ACD6 activity. However, despite these structural similarities, plants with hyperactive *ACD6* alleles and necrotic hybrids differ in their responses to temperature. While the immune phenotypes of plants with the Est-1 allele are largely insensitive to changes in temperature, the phenotypes of Mir-0×Se-0 hybrids and the *acd6*-1 mutant are attenuated at higher temperature (Figure S6), as is typical for immune responses [28].

### Complexity of hybrid necrosis in Mir-0×Se-0-like crosses

A notable feature of the Mir-0×Se-0 system is variation in the expression of hybrid necrosis. Variation in the degree of lethality has been documented in interspecfic *Drosophila* crosses as well [29], [30]. Different from these other systems, we have shown that phenotypic variation is primarily controlled by the strength and specificity of interactions between several sub-categories of Mir-0- and Se-0-like alleles.

Se-0-like alleles have a unique evolutionary history. The ancestral organization for this locus is likely to be the one found in the reference Col-0 strain, with two paralogs, At4g14390 and *ACD6*, derived from an ancient duplication event. Next, At4g14390 became pseudogenized, a state found in several Arabidopsis strains (Fig. S1A). This was followed by a tandem duplication creating the chimeric *ACD6A* gene upstream of *ACD6B*, which corresponds to *ACD6* in the reference genome (Figure 2A, Figure S3). This configuration is found in Bla-1, which has partial Se-0-like activity. Finally, the promoter and first exon of *ACD6B* was deleted, giving rise to the Se-0 allele, while the *ACD6B* copy of Bla-1 appears to have independently suffered a nonsense mutation. A more recent origin of the Se-0 allele compared to Bla-1 is also supported by their geographical distribution; while Bla-1 is broadly distributed at low frequency in Europe, Se-0 is found only in Northeast Spain, where it rose to high frequency.

It is interesting that the causal polymorphisms for hybrid necrosis in the *ACD6A* allele are derived from At4g14390. These polymorphisms could accumulate freely in At4g14390 once it became a pseudogene and therefore relieved from selective pressures. The duplication event that gave rise to *ACD6A* “resuscitated” part of this pseudogene and made these polymorphisms part of a functional gene again. The importance of pseudogenization and resurrection in determining the fate of duplicated genes has been proposed before [31], [32], but instances in which a contribution to functional divergence has been documented are rare ([33], [34]; see also [35]). Gene conversion involving pseudogenes is known to be a major source of immunoglobulin diversity in chicken [36], [37] and of antigenic variation in several human pathogens [38]

### Co-occurrence of Mir-0 and Se-0-like alleles in natural populations

Different from other examples of hybrid necrosis in Arabidopsis [11], [15], the Mir-0 and Se-0-like alleles are both locally common and can be found at high frequencies in the same population. In addition, we found individuals heterozygous for the causal alleles in nature. The high frequency of Mir-0- and Se-0-like alleles in the Costa Brava region is suggestive of a role in local adaptation. This interpretation is supported by the lack of polymorphisms found among Se-0-like alleles, possibly due to a recent selective sweep for this allele. The possibility that these alleles would have instead achieved high frequency in this area simply by genetic hitchhiking is not supported by our finding that both Mir-0- and Se-0-like alleles are present in several different genetic backgrounds (Figure 6).

**Figure 7:**
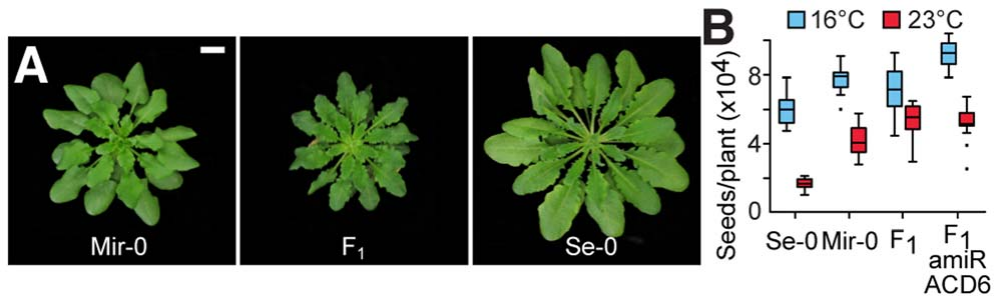
Effect of low temperature on hybrids. (**A**) Rosettes of plants grown for four weeks at 16°C in long days and then transferred to 4°C in short days for 16 weeks. Size bar = 1 cm. (**B**) Seed set for plants that were exposed to a six-week period of 4°C (short days) one week after sowing, and afterwards returned to the indicated temperatures. Averages from at least 15 individuals are shown. Seed set was significantly different between F_1_ and F_1_ amiR-ACD6 plants at 16°C (p<0.001), but not at 23°C (p = 0.60).

An alternative explanation for the patterns observed in Costa Brava is that Mir-0×Se-0 hybrids are conditionally advantageous or heterotic. Increased resistance to pathogens in these hybrids could compensate for their reduced fitness, especially in the mild climate of Northeast Spain, which would likely mitigate the severity of the necrosis (Figure S7). This hypothesis is partially supported by the observation that hybrids carrying both causal *ACD6* alleles could not be readily distinguished from inbred individuals in natural populations. It should be noted, however, that most plants in such population were small, probably due to abiotic stress exposure, possibly limiting the expression of hybrid necrosis symptoms (Figure 5C). Moreover, Mir-0×Se-0 hybrids can withstand long period of cold exposure without further reduction in their fitness, meaning that they would be able survive winter (most plants in Costa Brava seem to germinate in autumn and to overwinter as rosettes) (Figure 7). This situation would have similarities with what we observed for hyperactive Est-1 alleles of *ACD6*, which are maintained at intermediate frequencies by balancing selection [16]. Additional surveys of genetic diversity in the Costa Brava region would be required to test this hypothesis.

In conclusion, we have discovered a complex, single-gene hybrid necrosis system in which interactions between alleles with a diverse evolutionary history lead to different degrees of activation of the immune system. We have identified the causal polymorphisms and described the population genetic dynamics of the causal alleles. The complex structure and extraordinary functional diversity at the *ACD6* locus not only make it very similar to conventional immune receptors of the NLR class [7], [39], [40], but also point to a central role of *ACD6* in fine-tuning immunity in natural populations of Arabidopsis. Further investigation of this system will provide additional insight into the mechanism of *ACD6* action and will help to define the fuzzy boundary between beneficial priming of resistance and deleterious autoimmunity in plants.

## Materials and Methods

### Plant material and growth conditions

Seeds were obtained from the European Arabidopsis Stock Center (NASC). Some of the crosses have been described [11]. Plants were grown in growth rooms under long days (16 hours of light, 8 hours of dark) at 16°C with 65% humidity, unless stated otherwise.

### Genetic mapping

Genomic DNA was isolated from 96 Mir-0×Se-0 F_2_ plants with an F_1_-like phenotype using a BioSprint 96 (Qiagen, Hilden, Germany). Plants were genotyped using a panel of 149 SNPs [41] (Sequenom, San Diego, CA, USA). Fine mapping was performed using DNA from 864 additional necrotic F_2_ plants, isolated using a modification of the CTAB method for 96-well plates [42]. Markers used for fine-mapping are reported in Table S8. To test linkage of the necrosis phenotype in Bla-1×Hh-0 and ICE79×Bla-1, 96 F_2_ plants were genotyped for two markers flanking the *ACD6* region. In addition, 16 to 62 F_2_ plants each were grown from 36 crosses among accessions from the Costa Brava region, or between these accession and Se-0 or Mir-0, and presence of leaf necrosis was assessed (Table S3).

### Histology

Trypan blue (Sigma-Aldrich, St. Louis, MO, USA) staining was performed as described [43].

### Biomass and seed analysis

For dry weight analysis, the aerial parts of plants grown for six weeks at 16°C were dried at 85°C overnight, then weighed. For seed set analysis, 15 plants of each genotype were grown until the end of their life cycle in both 16°C and 23°C long days, with or without a six week vernalization treatment at 4°C in short days. Seeds were counted in six randomly chosen mature siliques per plant (at least 80 siliques per genotype under each condition). Total seed number for each plant was determined by multiplying seed averages per silique with the total number of siliques. The low seed production of Se-0 plants in 23°C conditions was due to a large fraction of aborted siliques. Tukey-Kramer tests were used to determine significance for multiple comparisons.

### *ACD6* sequence analysis

For Mir-0 and Hh-0, fosmid libraries were made using the Copy Control Fosmid Library production kit (Epicentre Biotechnologies, Madison, WI, USA) with 20 µg genomic DNA as starting material, following the manufacturer's instructions. The libraries were screened with two probes flanking the *ACD6* locus. Fosmids were shotgun sequenced and individual sequences were assembled using SeqMan (DNAstar, Madison, WI, USA). PCR fragments covering the entire region in Se-0 were amplified and sequenced. A similar approach was used to sequence the full-length *ACD6* gene from other accessions. Sequences were assembled using SeqMan and then aligned using CLUSTALW version 2 [44]. Neighbor joining trees were computed and plotted using MEGA5 [45].

### Transgenes

The amiRNA against *ACD6* has been described [16]. AmiRNAs targeting At4g14370 and At4g14390 were designed using the WMD online tool (http://wmd3.weigelworld.org; [17]; Table S9), and placed under control of the constitutive CaMV 35S promoter in pFK210 derived from [46]. All amiRNAs were transformed into Se-0 plants, and primary or later-generation transformants were crossed to Mir-0 or other accessions. The genomic fragments for Mir-0 and Hh-0 were PCR-amplified from fosmid clones. For the Se-0 genes in the *ACD6* region, genomic DNA was used as template for PCR amplification; to ensure specific amplification, it was necessary to amplify two different fragments each for *ACD6A* and *ACD6B*, and then reassemble each gene using specific restriction enzyme sites. Other *ACD6* alleles were similarly amplified from genomic DNA. Genomic constructs including the putative promoter region (from the 3′ end of the upstream gene) and several hundred base pairs of sequence beyond the putative 3′ UTR, were cloned into pFK202, a pGREEN-derived binary vector. Different regions were exchanged between alleles cloned into pFK202 using restriction enzymes; when restriction sites were not available, fragments were amplified from the two alleles, joined by overlap PCR and inserted into the appropriate genomic clone. Non-synonymous substitutions in the codons for amino acids 485, 486, 520 and 521 of the ACD6 protein, as well as a single leucine insertion between positions 482 and 483, were introduced by PCR-mediated mutagenesis into the Col-0 genomic construct [16]. Amino acid positions refer to the ACD6 protein sequence in the reference Col-0 strain, unless otherwise indicated. Constructs were introduced into plants by *Agrobacterium tumefaciens*-mediated transformation [47].

### Haplotype analysis

Genome-wide genotype information for 1,307 accessions was obtained from ref. [24], and haplotype similarity was visually assessed for a 60 kb region centered around the *ACD6* locus. The 120 accessions with the most similar haplotypes to known Mir-0-like alleles were divided into subgroups with identical or highly similar haplotypes, and one or two representative accessions from each subgroup were selected for further analysis. The sequence of the transmembrane region of *ACD6* was obtained for these accessions and compared to the sequences of known Mir-0-like alleles (Table S4). Test crosses were made for four of these accessions (Table S2).

### Expression assays

Quantitative reverse transcription PCR (RT-PCR) assays were performed as described [48], using RNA extracted from the 12^th^ leaf of 6-week old plants. Expression levels were normalized against *BETA-TUBULIN-2* (At5g62690). An experimentally quantified average amplification efficiency of 1.98 was used in the calculations [16]. Primers used for RT-PCR are given in Table S10.

### Field collections

An initial screening of *ACD6* sequences was performed on pooled leaf tissue collected from 27 sites in March 2012 in the Costa Brava region (Spain). One hundred seventy-nine pools (each from 6–10 plants) were assayed by PCR for the presence of Mir-0-like and Se-0-like sequences. Subsequently, approximately 2,200 samples were collected from the thirteen larger populations in the area. All samples were immediately frozen on dry ice to preserve their DNA. The region encoding the transmembrane domain of ACD6 (or ACD6B for Se-0-like accessions) was amplified by PCR and sequenced for all samples. Genotyping for the highly divergent KZ10-like alleles was performed using a previously described PCR-based assay [16].

### Sequencing and genotyping of multiplexed RAD-tag sequencing

For RAD-tag sequencing [49], genomic DNA from 2,112 wild individuals was quantified using a Qubit (Life Technologies, Carlsbad, CA, USA) and all samples were normalized to 20 ng/µl. Eleven RAD-seq libraries were prepared following the modifications described in [50], with double digestions using PstI and MseI restriction enzymes (Thermo Fisher Scientific, Waltham, MA, USA). The final eight PCR reactions per library were pooled and 250–500 bp fragments were selected by gel extraction. Libraries were sequenced on a HiSeq2000 instrument (Illumina, San Diego, CA, USA) with single-end 101 bp reads.

Reads were processed with SHORE (ver. 0.9; [51]) and mapped to the reference sequence, Col-0, with BWA [52], using the default parameters and allowing 5% mismatches. All mapped reads were converted into BAM format by Samtools (ver. 0.1.18; [53]) for further analysis. Single nucleotide polymorphism (SNP) genotypes were generated using the subprogram UnifiedGenotyper implemented in GATK (ver. 2.3–6; [54]) using default parameters. Only polymorphisms present as non-singletons in 80 fully sequenced Arabidopsis accessions [55] were accepted, to reduce the possibility of erroneously mapped markers, especially heterozygous markers. We removed markers with over 10% heterozygous calls, more than 20% missing information, or minor allele frequency (MAF) below 0.01. To generate the data matrix for subsequent analysis we used individuals that had been successfully typed for *ACD6* alleles, resulting in 1,688 samples and 3,641 markers.

### Population genetic analysis

Principle component analysis (PCA) was carried out using the adegenet package with 730 SNPs that had complete information (ver. 1.3–6; [56]) in R (http://www.R-project.org). To determine the number of unique genotypes we calculated the genetic distance based on the pairwise difference among samples using MEGA version 5 [45]. A sliding window approach was used to calculate F_st_ using the program HBKpermute (see [57]) implemented in ‘analysis’ built by the C++ class library, ‘libsequence’ [58]. To calculate F_st_, we used sliding windows of 10 SNPs, with 2 SNP steps. LD was estimated using the program ‘rsq’ implemented in ‘analysis’ [58]. LD decay was smoothened by estimating the least-square expectation of squared correlations (r^2^) from the nonlinear regression evaluation [59].

### Accession numbers

Sequences have been deposited in GenBank under accession numbers KC019116 to KC019169.

## Supporting Information

Figure S1 Analysis of candidate genes. (A) Rosettes of six-week-old plants. At4g14370 and At4g14400 (*ACD6*) were knocked down using amiRNAs. Size bar = 1 cm. (B) Relative expression levels of At4g14370 and *ACD6* in amiRNA plants, compared to parents and nontransgenic hybrids. Expression values are normalized to those of At4g14370 in Se-0 and of *ACD6B* in Mir-0. Averages from three biological replicates are reported. Error bars represent standard errors of the mean.
(TIF)

Figure S2 Se-0-like *ACD6* paralogs. (A) Hierarchical clustering of 17 Arabidopsis accessions and the *A. lyrata* MN47 strain based on At4g14390 sequence similarity (see Figure 2A). At4g14390 lacks a start codon in the strains boxed in dark blue. Se-0/Bla-1-like accessions are shown in orange (B) Expression analysis by RT-PCR of At4g14390, *ACD6A* and *ACD6B*. The PCR primers used to test expression of *ACD6B* were designed to amplify the *ACD6* sequence from Mir-0 as well.
(TIF)

Figure S3 Location of functional amino acid changes in the transmembrane domain of ACD6. Positions of the amino acid changes, insertions or regions that are causal for the altered activity of different *ACD6* alleles are indicated [16], [18] All amino acid positions refer to the Col-0 ACD6 protein; the corresponding positions on the Se-0 ACD6A sequence are given in parentheses.
(TIF)

Figure S4 Analysis of hybrids between accessions from the Costa Brava region. (A) Examples of six-week-old crosses between accessions from the Costa Brava region, and between these and Mir-0 and Se-0 plants (a Mir-0×Se-0 hybrid is shown for comparison). Size bar = 1 cm. (B) Relative expression levels of *PR1* in some of the crosses shown in panel A, and in their parents. Averages from three biological replicates are reported. Error bars represent standard errors.
(TIF)

Figure S5 Patterns of polymorphism in Costa Brava populations. (A) Comparison of patterns of LD decay around *ACD6* and across the genome in groups of individuals with different *ACD6* alleles. (B) F_st_ between different sub-groups of individuals in the Costa Brava population, defined by their *ACD6* allele type, using alleles with minor allele frequency of at least 0.2. Number of individuals in parentheses.
(TIF)

Figure S6 Influence of temperature shifts on *ACD6* activity. (A) Rosettes of four-week-old plants grown at 23°C in short days or at 16°C in long days. Short days were used for the 23°C experiments because under growth patterns under 23°C short days resembles more closely that of plants grown in long days at 16°C. (B) Plants grown for 18 days in 23°C long days and then moved to 16°C for four days. After the transfer, cell death throughout the plant was visible in transgenic lines expressing a hyper-active version of the Col-0 allele of *ACD6* (carrying either the *acd6*-1 mutation, L591F, or the two amino acid changes responsible for hyper-activation of the Est-1 allele, A566N and L634F). No or very mild increase in leaf necrosis was seen for plants transformed with the non-hyper-active Col-0 allele or with the original hyper-active Est-1 allele, which has additional substitutions compared to the two-amino-acid-swap construct. Exchanging the promoter region between the gACD6_Est1- and gACD6_A566N,L634F constructs did not alter their susceptibility to temperature. All transgenic lines were in the *acd6*-2 loss-of-function background. Size bars = 1 cm.
(TIF)

Figure S7 Temperature variation in the Costa Brava region of Spain. Light and medium tan represent the daily temperature range and 95% percentile range in the period 1973–2012. Dark tan shows the daily temperature range beginning in April of 2011, the year before sampling in this study, and the white line shows the daily mean temperature in the same period. Temperature information was obtained from the weather station of the Girona airport (Latitude 41.54.024°N, Longitude 2.45.537°E).
(TIF)

Table S1 Phenotypes of plants transformed with different transgenes.
(DOCX)

Table S2 Phenotypes of F_1_ individuals from crosses between Arabidopsis strains.
(DOCX)

Table S3 Segregation analysis for leaf necrosis in F_2_ populations.
(DOCX)

Table S4 Accessions predicted to carry a Mir-0- or Se-0-like allele of *ACD6* according to haplotype analyses.
(DOCX)

Table S5 Sampling sites along Costa Brava for the 2007 collection. and unique alleles initially identified in these populations.
(DOCX)

Table S6 Sampling sites along Costa Brava for the 2012 collection, and number of individuals collected at each site.
(DOCX)

Table S7 Comparison between *ACD6* alleles conferring increased immunity.
(DOCX)

Table S8 Markers on chromosome 4 used for fine mapping.
(DOCX)

Table S9 AmiRNA sequences.
(DOCX)

Table S10 Primers used for RT-PCR analyses.
(DOCX)
