# Supplementary figures and images for: Activation of the Arabidopsis thaliana Immune System by Combinations of Common ACD6 Alleles
Source: PLoS Genet. 2014 Jul 10;10(7):e1004459. doi: 10.1371/journal.pgen.1004459 (PMC4091793; doi:10.1371/journal.pgen.1004459)

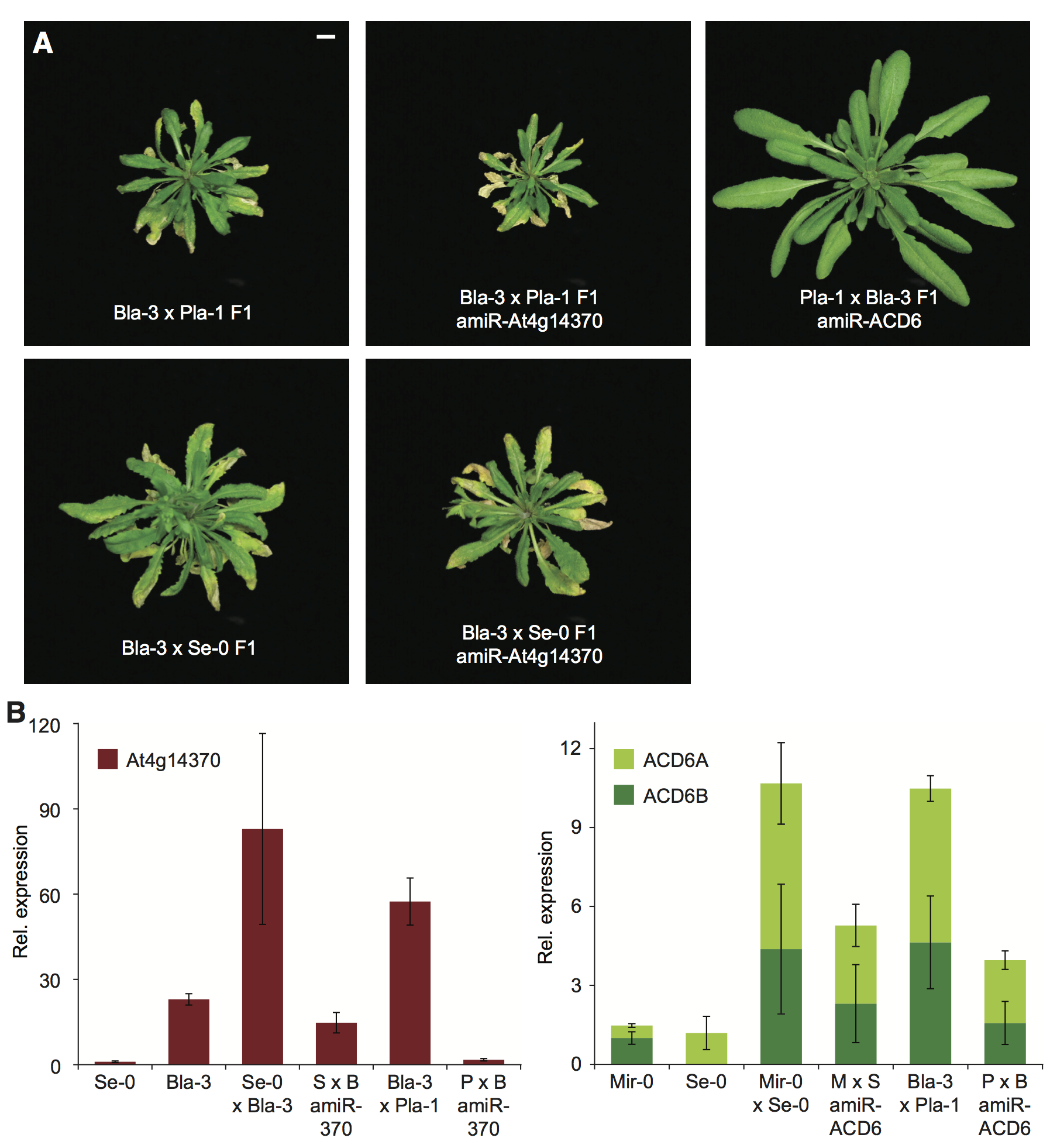

Supplement: Figure S1 — Analysis of candidate genes. (A) Rosettes of six-week-old plants. At4g14370 and At4g14400 (ACD6) were knocked down using amiRNAs. Size bar = 1 cm. (B) Relative expression levels of At4g14370 and ACD6 in amiRNA plants, compared to parents and nontransgenic hybrids. Expression values are normalized to those of At4g14370 in Se-0 and of ACD6B in Mir-0. Averages from three biological replicates are reported. Error bars represent standard errors of the mean. (TIF) [file pgen.1004459.s001.tif]

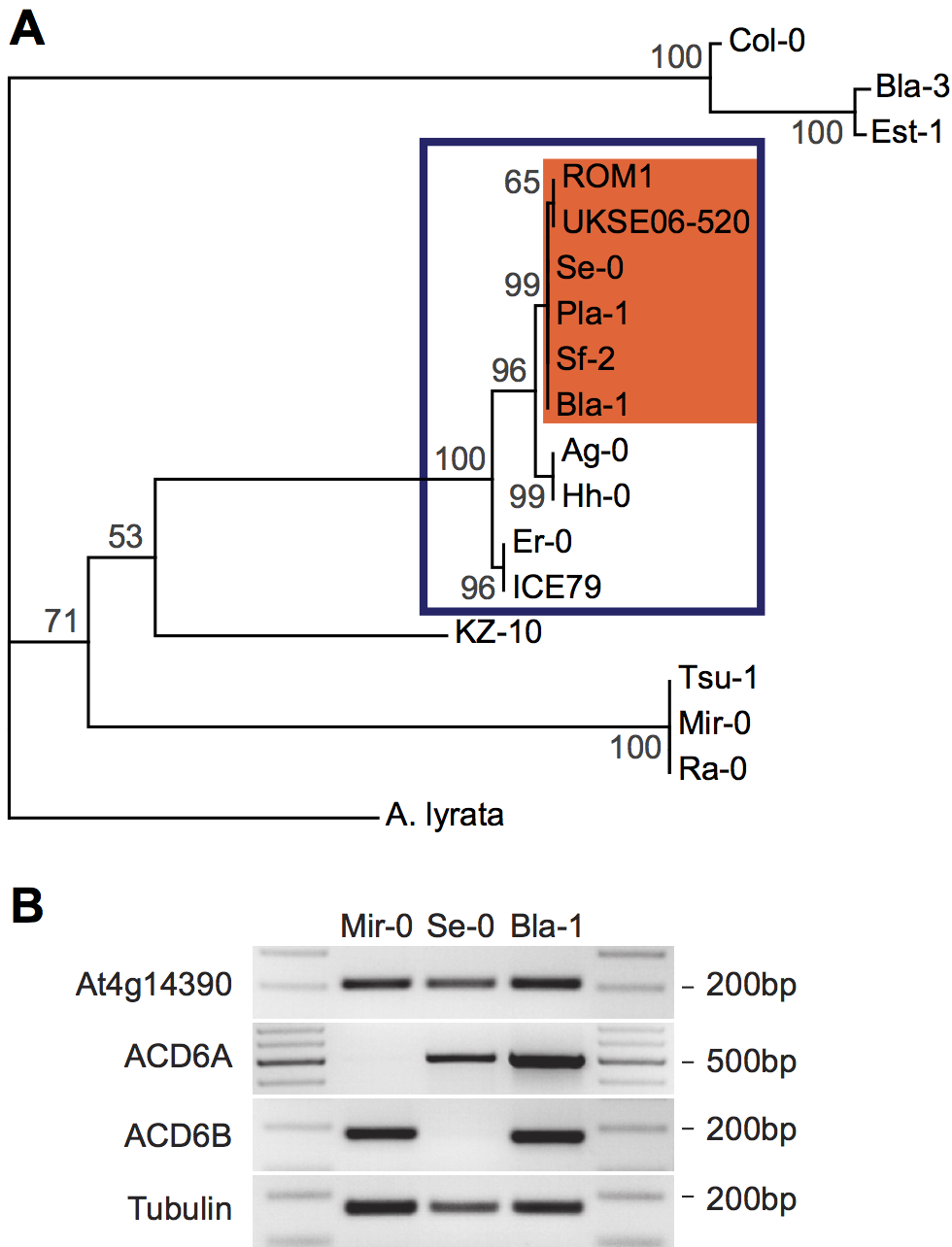

Supplement: Figure S2 — Se-0-like ACD6 paralogs. (A) Hierarchical clustering of 17 Arabidopsis accessions and the A. lyrata MN47 strain based on At4g14390 sequence similarity (see Figure 2A). At4g14390 lacks a start codon in the strains boxed in dark blue. Se-0/Bla-1-like accessions are shown in orange (B) Expression analysis by RT-PCR of At4g14390, ACD6A and ACD6B. The PCR primers used to test expression of ACD6B were designed to amplify the ACD6 sequence from Mir-0 as well. (TIF) [file pgen.1004459.s002.tif]

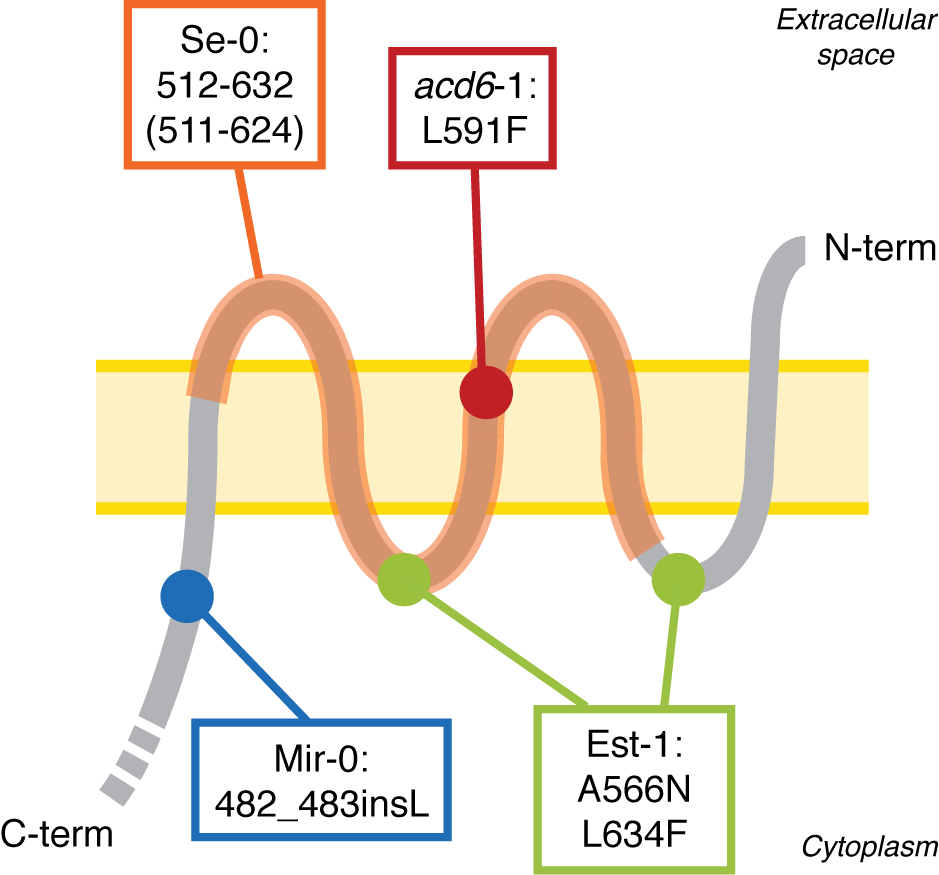

Supplement: Figure S3 — Location of functional amino acid changes in the transmembrane domain of ACD6. Positions of the amino acid changes, insertions or regions that are causal for the altered activity of different ACD6 alleles are indicated [16], [18] All amino acid positions refer to the Col-0 ACD6 protein; the corresponding positions on the Se-0 ACD6A sequence are given in parentheses. (TIF) [file pgen.1004459.s003.tif]

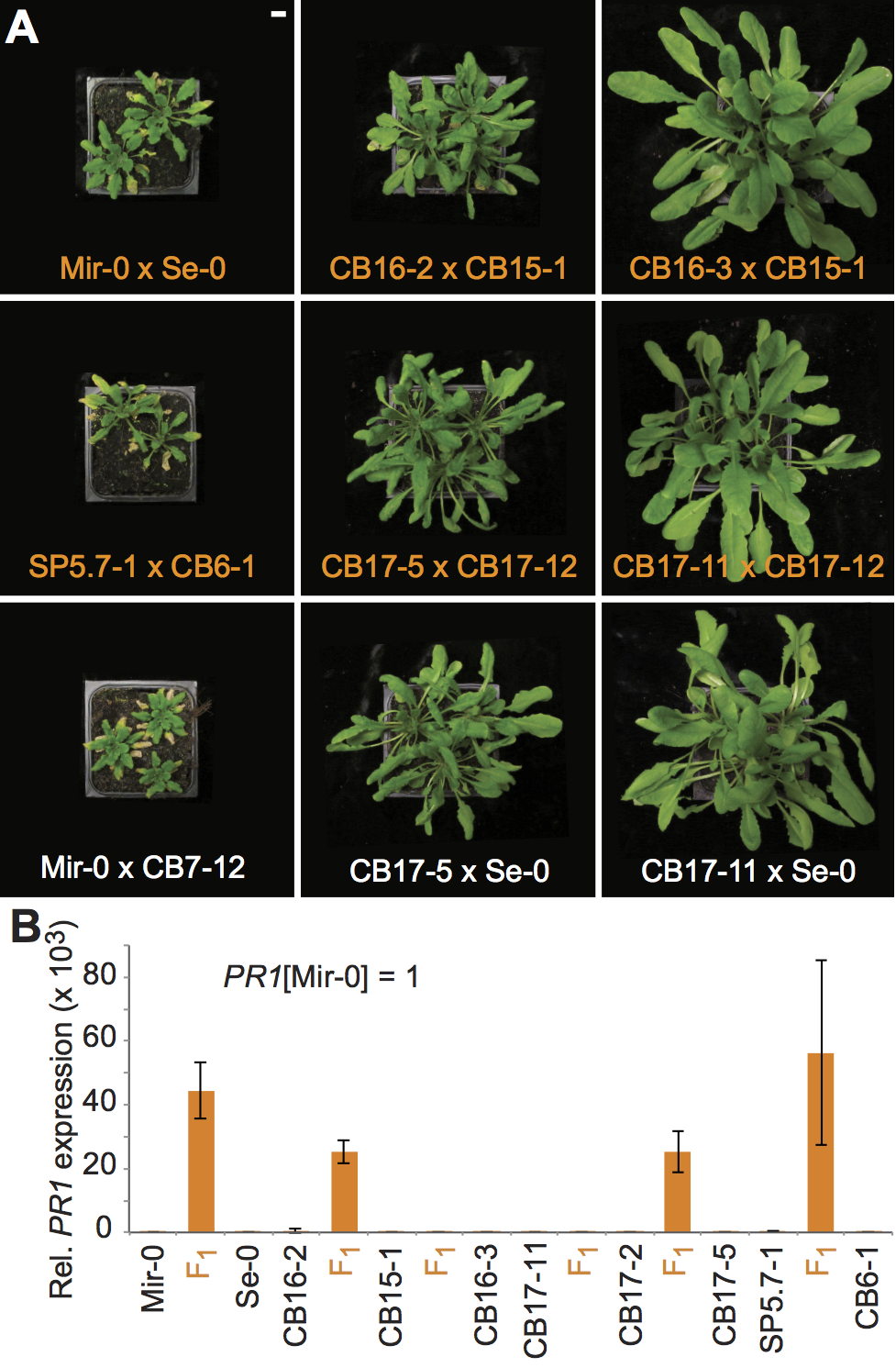

Supplement: Figure S4 — Analysis of hybrids between accessions from the Costa Brava region. (A) Examples of six-week-old crosses between accessions from the Costa Brava region, and between these and Mir-0 and Se-0 plants (a Mir-0×Se-0 hybrid is shown for comparison). Size bar = 1 cm. (B) Relative expression levels of PR1 in some of the crosses shown in panel A, and in their parents. Averages from three biological replicates are reported. Error bars represent standard errors. (TIF) [file pgen.1004459.s004.tif]

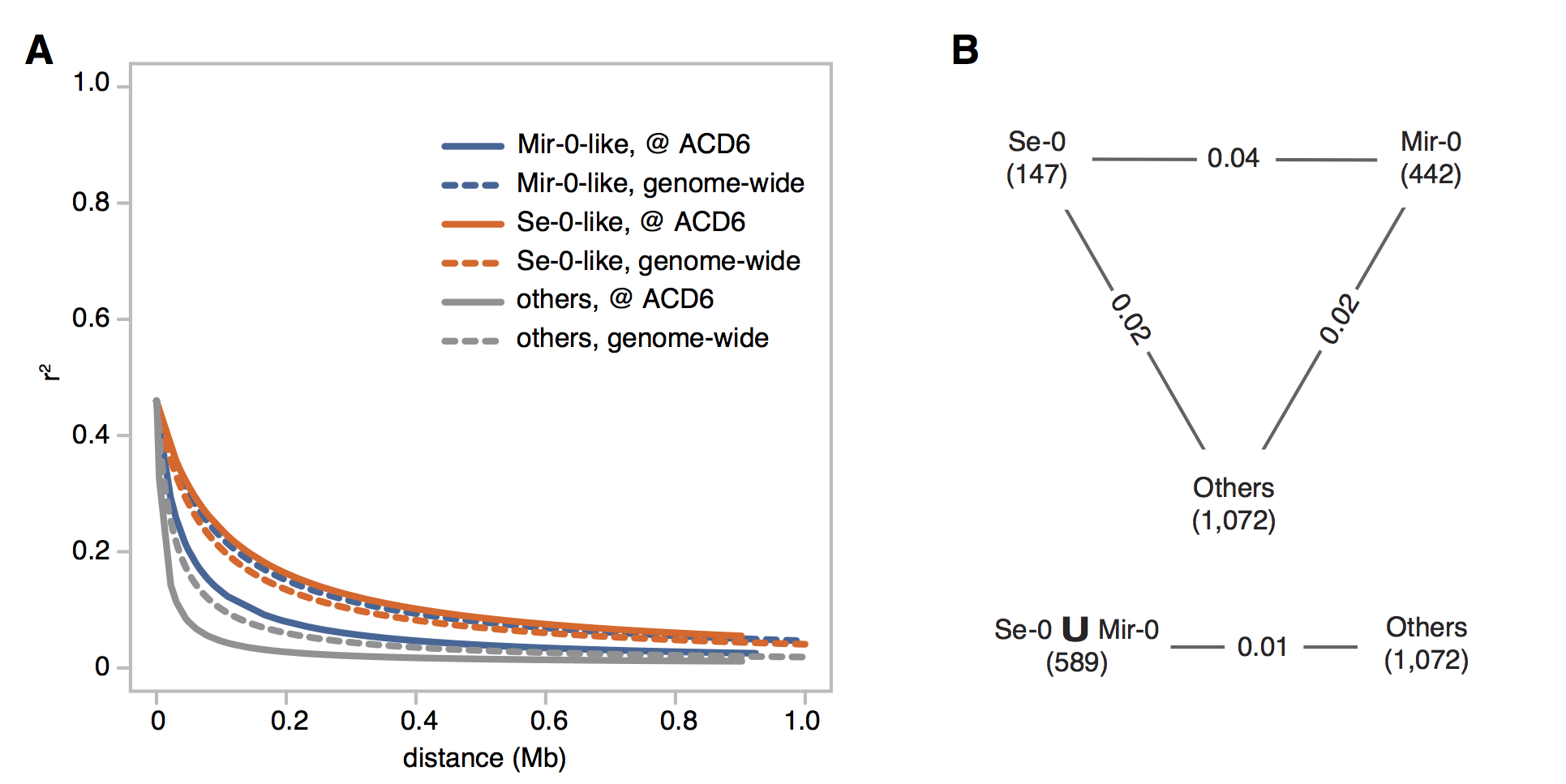

Supplement: Figure S5 — Patterns of polymorphism in Costa Brava populations. (A) Comparison of patterns of LD decay around ACD6 and across the genome in groups of individuals with different ACD6 alleles. (B) Fst between different sub-groups of individuals in the Costa Brava population, defined by their ACD6 allele type, using alleles with minor allele frequency of at least 0.2. Number of individuals in parentheses. (TIF) [file pgen.1004459.s005.tif]

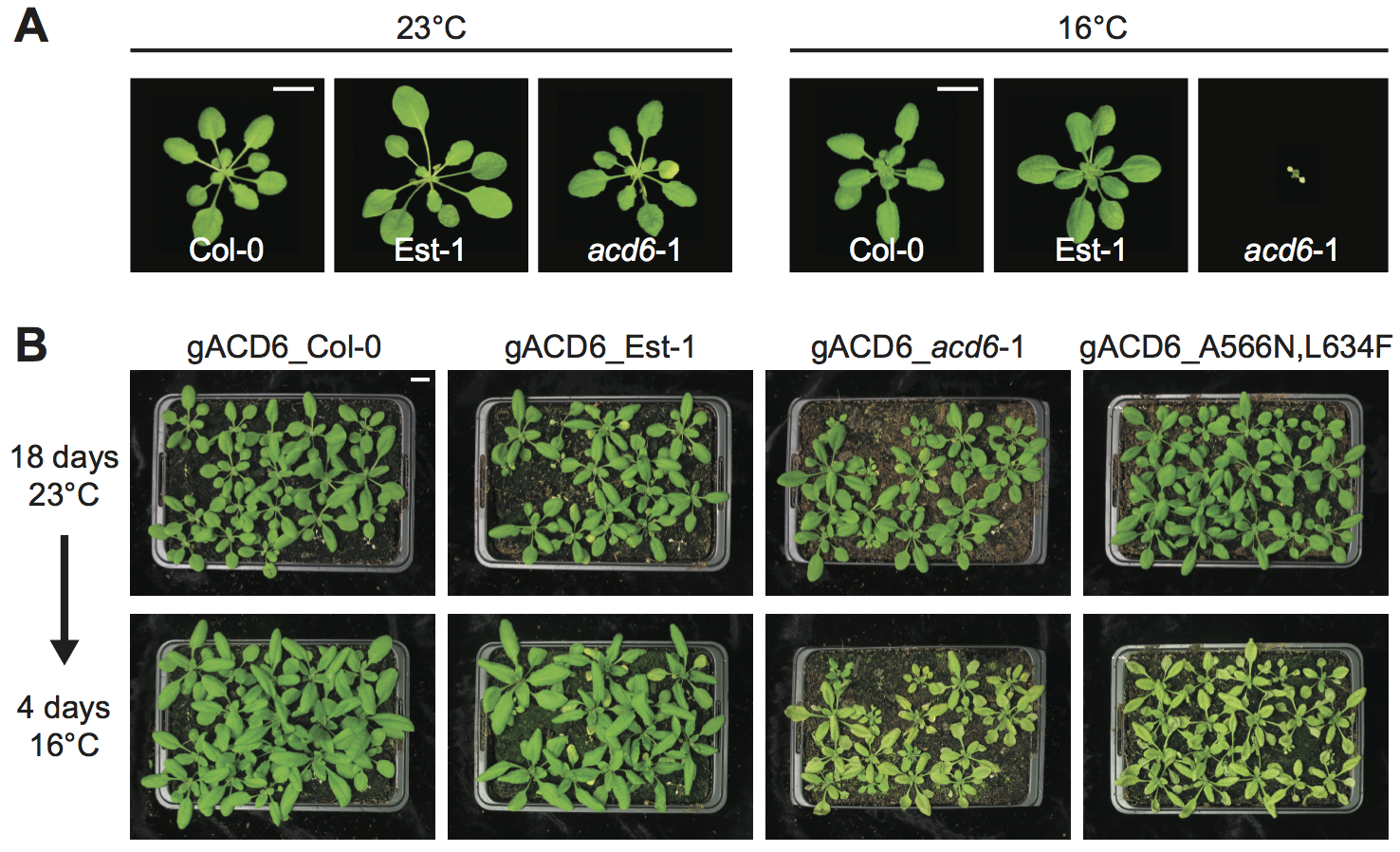

Supplement: Figure S6 — Influence of temperature shifts on ACD6 activity. (A) Rosettes of four-week-old plants grown at 23°C in short days or at 16°C in long days. Short days were used for the 23°C experiments because under growth patterns under 23°C short days resembles more closely that of plants grown in long days at 16°C. (B) Plants grown for 18 days in 23°C long days and then moved to 16°C for four days. After the transfer, cell death throughout the plant was visible in transgenic lines expressing a hyper-active version of the Col-0 allele of ACD6 (carrying either the acd6-1 mutation, L591F, or the two amino acid changes responsible for hyper-activation of the Est-1 allele, A566N and L634F). No or very mild increase in leaf necrosis was seen for plants transformed with the non-hyper-active Col-0 allele or with the original hyper-active Est-1 allele, which has additional substitutions compared to the two-amino-acid-swap construct. Exchanging the promoter region between the gACD6_Est1- and gACD6_A566N,L634F constructs did not alter their susceptibility to temperature. All transgenic lines were in the acd6-2 loss-of-function background. Size bars = 1 cm. (TIF) [file pgen.1004459.s006.tif]

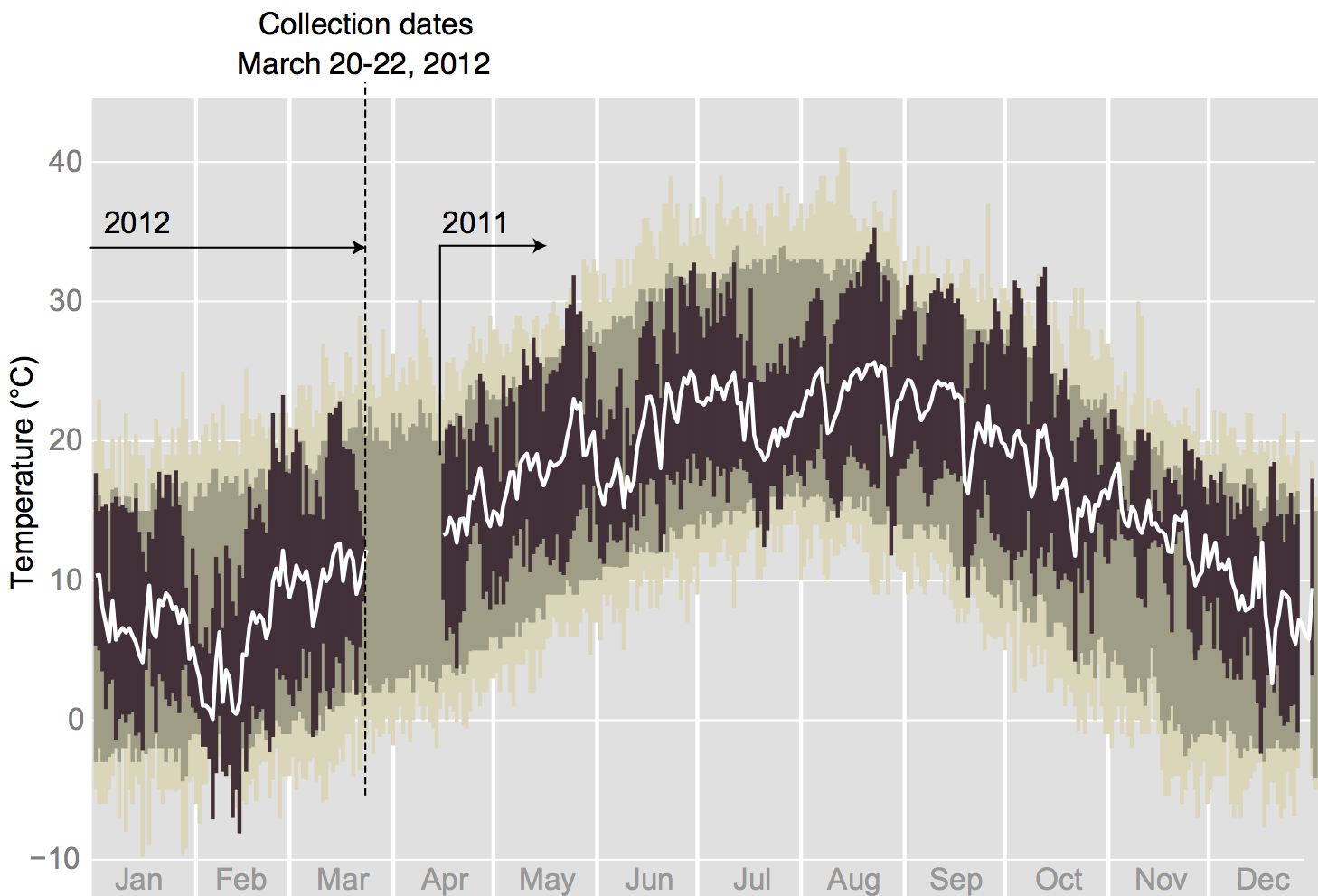

Supplement: Figure S7 — Temperature variation in the Costa Brava region of Spain. Light and medium tan represent the daily temperature range and 95% percentile range in the period 1973–2012. Dark tan shows the daily temperature range beginning in April of 2011, the year before sampling in this study, and the white line shows the daily mean temperature in the same period. Temperature information was obtained from the weather station of the Girona airport (Latitude 41.54.024°N, Longitude 2.45.537°E). (TIF) [file pgen.1004459.s007.tif]
