# Supplementary material for: Activation of the Arabidopsis thaliana Immune System by Combinations of Common ACD6 Alleles
Source: PLoS Genet. 2014 Jul 10;10(7):e1004459. doi: 10.1371/journal.pgen.1004459 (PMC4091793; doi:10.1371/journal.pgen.1004459)
Supplement: Table S1 — Phenotypes of plants transformed with different transgenes. (DOCX) [file pgen.1004459.s008.docx]

**Table S1.** **Phenotypes of plants transformed with different transgenes.** Purple indicates severe, orange mild, green no necrosis, and grey not assessed.

|  | **Accessions** | Col-0 | Mir-0 | Hh-0 | Se-0 | Bla-1 | Ag-0 | Belmonte4-94 | C24 | CIBC5 | Got-7 | Kin-0 | Lm-2 | Ma-0 | Mt-0 | N4 | Omo2-1 | Oy-0 | Ra-0 | Ren-1 | ROM-1 | Sq-8 | Shahdara | Sü-0 | TDr-1 | Ts-5 | TOU-A1-96 | UKSE06-520 | Van-0 | WAR | Ws | Ws-0 | Yo-0 |
| --- | --- | --- | --- | --- | --- | --- | --- | --- | --- | --- | --- | --- | --- | --- | --- | --- | --- | --- | --- | --- | --- | --- | --- | --- | --- | --- | --- | --- | --- | --- | --- | --- | --- |
| **Transgenes** | gACD6_Mir-0 |  |  |  |  |  |  |  |  |  |  |  |  |  |  |  |  |  |  |  |  |  |  |  |  |  |  |  |  |  |  |  |  |
|  | gACD6_Hh-0 |  |  |  |  |  |  |  |  |  |  |  |  |  |  |  |  |  |  |  |  |  |  |  |  |  |  |  |  |  |  |  |  |
|  | gACD6A_C24 |  |  |  |  |  |  |  |  |  |  |  |  |  |  |  |  |  |  |  |  |  |  |  |  |  |  |  |  |  |  |  |  |
|  | gACD6A_CB17-5 |  |  |  |  |  |  |  |  |  |  |  |  |  |  |  |  |  |  |  |  |  |  |  |  |  |  |  |  |  |  |  |  |
|  | gAt4g14390_Se-0 |  |  |  |  |  |  |  |  |  |  |  |  |  |  |  |  |  |  |  |  |  |  |  |  |  |  |  |  |  |  |  |  |
|  | gACD6A_Se-0 |  |  |  |  |  |  |  |  |  |  |  |  |  |  |  |  |  |  |  |  |  |  |  |  |  |  |  |  |  |  |  |  |
|  | gACD6B_Se-0 |  |  |  |  |  |  |  |  |  |  |  |  |  |  |  |  |  |  |  |  |  |  |  |  |  |  |  |  |  |  |  |  |
|  | gACD6A_Bla-1 |  |  |  |  |  |  |  |  |  |  |  |  |  |  |  |  |  |  |  |  |  |  |  |  |  |  |  |  |  |  |  |  |
|  | gACD6_Col-0 |  |  |  |  |  |  |  |  |  |  |  |  |  |  |  |  |  |  |  |  |  |  |  |  |  |  |  |  |  |  |  |  |
