# Supplementary material for: Activation of the Arabidopsis thaliana Immune System by Combinations of Common ACD6 Alleles
Source: PLoS Genet. 2014 Jul 10;10(7):e1004459. doi: 10.1371/journal.pgen.1004459 (PMC4091793; doi:10.1371/journal.pgen.1004459)
Supplement: Table S3 — Segregation analysis for leaf necrosis in F2 populations. (DOCX) [file pgen.1004459.s010.docx]

**Table S3. Segregation analysis for leaf necrosis in F_2_ populations.**

| **Maternal**  **accession** | **Paternal**  **accession** | **Individuals with necrosis/ total** | | **Fraction** |
| --- | --- | --- | --- | --- |
| Mir-0 | CB15-1 | 13/30 | 0.43 | |
| Se-0 | CB3-1 | 0/41 | 0 | |
| CB2-1 | Se-0 | 0/28 | 0 | |
| CB5-1 | Se-0 | 0/32 | 0 | |
| CB5-1 | CB6-1 | 0/28 | 0 | |
| CB5-4 | CB3-1 | 0/30 | 0 | |
| CB5-4 | CB17-2 | 0/31 | 0 | |
| CB5-4 | CB17-11 | 0/31 | 0 | |
| CB5-4 | CB21.1-1 | 0/31 | 0 | |
| CB13-2 | Se-0 | 0/44 | 0 | |
| CB13-2 | CB5-4 | 0/31 | 0 | |
| CB13-2 | CB6-1 | 0/62 | 0 | |
| CB13-2 | CB22-3 | 0/60 | 0 | |
| CB15-1 | CB17-2 | 0/30 | 0 | |
| CB16-2 | Se-0 | 11/32 | 0.34 | |
| CB16-2 | CB5-4 | 0/30 | 0 | |
| CB16-2 | CB6-1 | 16/33 | 0.48 | |
| CB16-2 | CB15-1 | 16/30 | 0.53 | |
| CB16-2 | CB22-3 | 18/32 | 0.56 | |
| CB16-3 | Se-0 | 0/29 | 0 | |
| CB16-3 | CB5-4 | 0/32 | 0 | |
| CB16-3 | CB6-1 | 0/32 | 0 | |
| CB16-3 | CB22-3 | 0/32 | 0 | |
| CB17-5 | Se-0 | 33/62 | 0.53 | |
| CB17-5 | CB5-4 | 0/62 | 0 | |
| CB17-5 | CB15-1 | 32/60 | 0.53 | |
| CB17-5 | CB17-12 | 8/16 | 0.50 | |
| CB17-8 | Se-0 | 0/30 | 0 | |
| CB17-11 | Se-0 | 0/46 | 0 | |
| CB17-11 | CB15-1 | 0/31 | 0 | |
| CB17-11 | CB17-12 | 0/30 | 0 | |
| CB17-11 | CB22-3 | 0/31 | 0 | |
| CB17-13 | Se-0 | 0/29 | 0 | |
| CB17-13 | CB15-1 | 0/31 | 0 | |
| CB21.1-1 | CB15-1 | 16/31 | 0.52 | |
| CB22-3 | CB3-1 | 0/30 | 0 | |
