# Supplementary material for: Activation of the Arabidopsis thaliana Immune System by Combinations of Common ACD6 Alleles
Source: PLoS Genet. 2014 Jul 10;10(7):e1004459. doi: 10.1371/journal.pgen.1004459 (PMC4091793; doi:10.1371/journal.pgen.1004459)
Supplement: Table S4 — Accessions predicted to carry a Mir-0- or Se-0-like allele of ACD6 according to haplotype analyses. (DOCX) [file pgen.1004459.s011.docx]

**Table S4. Accessions predicted to carry a Mir-0- or Se-0-like allele of *ACD6* according to haplotype analyses.** Only accessions for which the sequence of the transmembrane domain of *ACD6* (or *ACD6B*) as Mir-0- or Se-0-like could be confirmed are reported.

| **Accession** | **Origin** | **Most similar *ACD6* allele** |
| --- | --- | --- |
| Belmonte-4-94 | Italy | Mir-0 |
| Brö 1-6 | South Sweden | Mir-0 |
| Dra-2 | South Sweden | Mir-0 |
| Fri-3 | South Sweden | Mir-0 |
| Li-3 | Germany | Mir-0 |
| NC-1 | France | Mir-0 |
| TDr-3 | South Sweden | Mir-0 |
| TIV-1 | Italy | Mir-0 |
| TOU-A1-98 | France | Mir-0 |
| UKSW06-280 | UK | Mir-0 |
| Fjä 1-5 | South Sweden | Er-0 |
| Fjä 2-4 | South Sweden | Er-0 |
| NFC-20 | UK | Er-0 |
| TDr-8 | South Sweden | Er-0 |
| T690 | South Sweden | Er-0 |
| T1050 | South Sweden | Er-0 |
| CAM-58 | France | Hh-0 |
| CS22538 | Netherlands | Hh-0 |
| DraIV 6-1 | Czech Republic | Hh-0 |
| LAC-3 | France | Hh-0 |
| Mh-0 | Poland | Hh-0 |
| TDr-1 | South Sweden | Hh-0 |
| TDr-7 | South Sweden | Hh-0 |
| TDr-9 | South Sweden | Hh-0 |
| TOU-A1-96 | France | Hh-0 |
| DAM 1 | France | Bla-1 |
| ROM-1 | France | Bla-1 |
| UKID77/Sü-1 | UK | Bla-1 |
| UKSE06-520 | UK | Bla-1 |
