# Supplementary material for: Activation of the Arabidopsis thaliana Immune System by Combinations of Common ACD6 Alleles
Source: PLoS Genet. 2014 Jul 10;10(7):e1004459. doi: 10.1371/journal.pgen.1004459 (PMC4091793; doi:10.1371/journal.pgen.1004459)
Supplement: Table S5 — Sampling sites along Costa Brava for the 2007 collection. and unique alleles initially identified in these populations. (DOCX) [file pgen.1004459.s012.docx]

**Table S5. Sampling sites along Costa Brava for the 2007 collection, and unique alleles initially identified in these populations.**

| **Site** | **Town** | **Latitude (°N)** | **Longitude (°E)** | **Alleles** |
| --- | --- | --- | --- | --- |
| pCB1 | La Montgoda | 41.42.645 | 2.52.579 | CB1-1 |
| pCB2 | ﻿Salionç | 41.44.565 | 2.57.509 | CB2-1 |
| pCB3 | Platja d'Aro | 41.48.847 | 3.03.813 | CB3-1; CB3-2; CB3-3 |
| pCB4 | Platja d'Aro | 41.48.577 | 3.03.439 | CB4-2 |
| pCB5 | Platja d'Aro | 41.48.616 | 3.03.391 | CB5-1; CB5-2; CB5-3; CB5-4 |
| pCB6 | Platja d'Aro | 41.48.598 | 3.03.366 | CB6-1 |
| pCB7 | Tossa de Mar | 41.43.482 | 2.55.622 | CB7-2 |
| pCB8 | Tossa de Mar | 41.43.528 | 2.55.645 | CB8 |
| pCB9 | Tossa de Mar | 41.43.601 | 2.55.594 | CB9-1 |
| pCB10 | Tossa de Mar | 41.43.684 | 2.55.555 | CB10-1; CB10-2 |
| pCB11 | Tossa de Mar | 41.43.709 | 2.55.242 | CB11-1 |
| pCB12 | Tossa de Mar | 41.43.488 | 2.55.574 |  |
| pCB13 | Tossa de Mar | 41.43.010 | 2.55.916 | CB13-2 |
| pCB14 | Llagostera | 41.49.750 | 2.53.515 | CB14 |
| pCB15 | Llagostera | 41.50.082 | 2.53.431 | CB15-1 |
| pCB16 | Llagostera | 41.49.561 | 2.53.277 | CB16-1; CB16-2; CB16-3; CB16-4 |
| pCB16.1 | Llagostera | 41.49.665 | 2.53.215 | CB16.1-1; CB16.1-3 |
| pCB17 | Llagostera | 41.49.328 | 2.53.615 | CB17-8; CB17-10; CB17-11; CB17-12; CB17-13 |
| pCB18 | Cassà de la Selva | 41.54.145 | 2.51.477 | CB18-2 |
| pCB19 | Llambilles | 41.55.254 | 2.52.062 | CB19-1; CB19-5 |
| pCB20 | Sant Gregori | 41.59.374 | 2.45.654 | CB20-1 |
| pCB21 | San Eloi | 41.43.756 | 2.54.420 | CB21-1 |
| pCB21.1 | San Eloi | 41.43.765 | 2.54.517 | CB21.1-1 |
| pCB22 | Lloret de Mar | 41.42.333 | 2.51.600 | CB22-3; CB22-5 |
| pSP1 | Sant Celoni | 41.40.196 | 2.28.560 | SP1-8.2; SP1-13.2; SP1-15.1 |
| pSP5 | Sant Celoni | 41.40.112 | 2.28.536 | SP5-5.2; SP5-7.1; SP5-14.2 |
| pSP6 | Can Pigdemir | 41.39.247 | 2.29.162 | SP6-1.2; SP6-2.1; SP6-7.1; SP6-8.1; SP6-13.1 |
| pSP8 | Can Pigdemir | 41.38.554 | 2.30.010 | SP8-1.2; SP8-7.2 |
