# Supplementary material for: Activation of the Arabidopsis thaliana Immune System by Combinations of Common ACD6 Alleles
Source: PLoS Genet. 2014 Jul 10;10(7):e1004459. doi: 10.1371/journal.pgen.1004459 (PMC4091793; doi:10.1371/journal.pgen.1004459)
Supplement: Table S6 — Sampling sites along Costa Brava for the 2012 collection, and number of individuals collected at each site. (DOCX) [file pgen.1004459.s013.docx]

**Table S6. Sampling sites along Costa Brava for the 2012 collection, and number of individuals collected at each site.**

| **Site** | **Town** | **Latitude (°N)** | **Longitude (°E)** | **N. of individuals collected** | |
| --- | --- | --- | --- | --- | --- |
| pCB5.4 | Platja d'Aro | 41.48.456 | 3.03.345 | 19 |  |
| pCB5.5 | Platja d'Aro | 41.48.427 | 3.03.183 | 461 |  |
| pCB13 | Tossa de Mar | 41.43.048 | 2.55.900 | 56 |  |
| pCB15.9 | Llagostera | 41.50.152 | 2.53.430 | 124 |  |
| pCB16 | Llagostera | 41.49.543 | 2.53.272 | 640 |  |
| pCB16.1 | Llagostera | 41.49.693 | 2.53.203 | 176 |  |
| pCB16.4 | Llagostera | 41.49.582 | 2.53.337 | 48 |  |
| pCB16.5 | Llagostera | 41.49.756 | 2.53.230 | 68 |  |
| pCB21 | San Eloi | 41.43.713 | 2.54.491 | 132 |  |
| pCB21.1 | San Eloi | 41.43.760 | 2.54.532 | 136 |  |
| pCB21.2 | San Eloi | 41.43.799 | 2.54.580 | 132 |  |
| pCB21.9 | San Eloi | 41.43.721 | 2.54.457 | 16 |  |
| pSP5 | Sant Celoni | 41.40.182 | 2.28.897 | 192 |  |
