# Supplementary material for: Activation of the Arabidopsis thaliana Immune System by Combinations of Common ACD6 Alleles
Source: PLoS Genet. 2014 Jul 10;10(7):e1004459. doi: 10.1371/journal.pgen.1004459 (PMC4091793; doi:10.1371/journal.pgen.1004459)
Supplement: Table S7 — Comparison between ACD6 alleles conferring increased immunity. (DOCX) [file pgen.1004459.s014.docx]

**Table S7. Comparison between *ACD6* alleles conferring increased immunity.**

|  | **Increases immunity** | **Temp. sens.?** | **Phenotype at 16°C** | **Phenotype at 23°C** | **Causal polymorphism** | **Distribution** |
| --- | --- | --- | --- | --- | --- | --- |
| ***acd6*-1** | alone | yes | dead | necrosis/ severe necrosis | single aa substitution in TM domain (L591F) | (artificial, EMS mutant) |
| **Est-1** | alone | no | necrosis | necrosis | two aa substitutions in TM domain (A566N and L634F) | ~20% frequency in both local and regional populations |
| **Mir-0** | in combination with Se-0 allele | yes | in hybrids: severe necrosis | in hybrids: no developmental defects | one aa insertion in TM domain (482_483insL) | ~6% frequency in the global Arabidopsis population; at ~22% in the Costa Brava |
| **Se-0** | in combination with Mir-0 allele | yes |  |  | polymorphisms in the TM domain (between aa 512-634) | found exclusively in the Costa Brava, at ~9% frequency |
