# Supplementary material for: Activation of the Arabidopsis thaliana Immune System by Combinations of Common ACD6 Alleles
Source: PLoS Genet. 2014 Jul 10;10(7):e1004459. doi: 10.1371/journal.pgen.1004459 (PMC4091793; doi:10.1371/journal.pgen.1004459)
Supplement: Table S8 — Markers on chromosome 4 used for fine mapping. (DOCX) [file pgen.1004459.s015.docx]

**Table S8. Markers on chromosome 4 used for mapping.**

| **Position (Mb)** | **Forward primer** | **Reverse primer** | **Marker type** | **Restriction enzyme** |
| --- | --- | --- | --- | --- |
| 7.98 | GGAGCAAATGGAATCCTTCA | TCTTACCACCTCGGTTTTCG | CAPS | ScrFI |
| 8.00 | ACATGTCCCAAGCACACAAA | TGGTGAGGCAATTGCTAAAA | CAPS | ScrFI |
| 8.02* | CTTGGCTGTGAAACGGTCTT | CACCAAGCTTCTGCAAATGA | CAPS | HaeIII |
| 8.11 | GCAAGAGCCTTGTTTCATCC | CGGTGAGCTTCTTCTGGAAC | CAPS | DdeI |
| 8.27 | AGAAGGGGTTGGATCTCGTT | CCAGGGGCATGATTGATACT | CAPS | XmnI |
| 8.27 | TCATGGACTTTGGACCTATCC | TTGCAGATGGGTAGTTGTCG | CAPS | MaeIII |
| 8.31 | TGGATGGTGCTACTGATCCTT | GATCGTTCCCGACATTATCC | Sequencing |  |
| 8.37 | AGCTCGGAAACCGGTAATCT | CGGTATGGAGCCGAGAATAA | Sequencing |  |
| 8.54 | GGTTCTCATTCAACGACTTTCC | TTGGGGAGTTAAGGGATCAA | CAPS | BsmAI |
