# Supplementary material for: Activation of the Arabidopsis thaliana Immune System by Combinations of Common ACD6 Alleles
Source: PLoS Genet. 2014 Jul 10;10(7):e1004459. doi: 10.1371/journal.pgen.1004459 (PMC4091793; doi:10.1371/journal.pgen.1004459)
Supplement: Table S9 — AmiRNA sequences. (DOCX) [file pgen.1004459.s016.docx]

**Table S9. AmiRNA sequences.**

| **Gene** | **AmiRNA sequences** |
| --- | --- |
| At4g14370 | TAAGTATCAATCATGCCCGTG |
|  | TTGAGCGAAATTTAGGCCCTC |
|  | TAATGTATGACCACACGCGGT |
| At4g14390 | TCAAGTTAGACTTCTTCCGTT |
|  | TTATACTGCATAAAAGTCGCG |
| At4g14400 (*ACD6*) | TTAATGGTGACTAAAGGCCGT |
