# Supplementary material for: Activation of the Arabidopsis thaliana Immune System by Combinations of Common ACD6 Alleles
Source: PLoS Genet. 2014 Jul 10;10(7):e1004459. doi: 10.1371/journal.pgen.1004459 (PMC4091793; doi:10.1371/journal.pgen.1004459)
Supplement: Table S10 — Primers used for RT-PCR analyses. (DOCX) [file pgen.1004459.s017.docx]

**Table S10. Primers used for RT-PCR analyses.**

| **Gene** | **Forward primer** | **Reverse primer** | **Product size (bp)** |
| --- | --- | --- | --- |
| *ACD6* | GAGATGGCTACCTGTCTGGTG | CTCTTGGCCTTCAAAGCAAC | 233 |
| *ACD6A* | TGTCTGAAGAAGTTGAGAAGCA | CACGGTCCACGTTGTCGTCT | 502 |
| *ACD6B* | GTTTCGAATCTGTTAAGTCA | TGCCCAAGTTTGGTTTGTTG | 177 |
| At4g14390 | TACGGAGCATCCATTGGGTA | CGTGGAGAACATTCTGACCA | 205 |
| *PR1* | CGTTCACATAATTCCCACGA | AAGAGGCAACTGCAGACTCA | 275 |
| *β-tubulin-2* | GAGCCTTACAACGCTACTCTGTCTGTC | ACACCAGACATAGTAGCAGAAATCAAG | 167 |
